# Fortuitously compatible protein surfaces primed allosteric control in cyanobacterial photoprotection

**DOI:** 10.1038/s41559-023-02018-8

**Published:** 2023-04-03

**Authors:** Niklas Steube, Marcus Moldenhauer, Paul Weiland, Dominik Saman, Alexandra Kilb, Adán A. Ramírez Rojas, Sriram G. Garg, Daniel Schindler, Peter L. Graumann, Justin L. P. Benesch, Gert Bange, Thomas Friedrich, Georg K. A. Hochberg

**Affiliations:** 1 Max Planck Institute for Terrestrial Microbiology, Marburg, Germany; 2 Institute of Chemistry PC14, Technische Universität Berlin, Berlin, Germany; 3 Department of Chemistry, University of Marburg, Marburg, Germany; 4 Center for Synthetic Microbiology (SYNMIKRO), Marburg, Germany; 5 Department of Chemistry, Oxford University, Oxford, UK; 6 Kavli Institute for Nanoscience Discovery, Oxford University, Oxford, UK

**Keywords:** Molecular evolution, Modularity

## Abstract

Highly specific interactions between proteins are a fundamental prerequisite for life, but how they evolve remains an unsolved problem. In particular, interactions between initially unrelated proteins require that they evolve matching surfaces. It is unclear whether such surface compatibilities can only be built by selection in small incremental steps, or whether they can also emerge fortuitously. Here, we used molecular phylogenetics, ancestral sequence reconstruction and biophysical characterization of resurrected proteins to retrace the evolution of an allosteric interaction between two proteins that act in the cyanobacterial photoprotection system. We show that this interaction between the orange carotenoid protein (OCP) and its unrelated regulator, the fluorescence recovery protein (FRP), evolved when a precursor of FRP was horizontally acquired by cyanobacteria. FRP’s precursors could already interact with and regulate OCP even before these proteins first encountered each other in an ancestral cyanobacterium. The OCP–FRP interaction exploits an ancient dimer interface in OCP, which also predates the recruitment of FRP into the photoprotection system. Together, our work shows how evolution can fashion complex regulatory systems easily out of pre-existing components.

## Abstract (web summary)

Molecular phylogenetics, ancestral sequence reconstruction and biophysical protein characterization are used to investigate the interaction between the orange carotenoid protein and its unrelated regulator, the fluorescence recovery protein (FRP). This interaction evolved when a precursor of FRP was horizontally acquired by cyanobacteria.

## Main

Allosteric interactions between proteins are a ubiquitous form of biochemical regulation in which the active site of one protein is affected by binding of another protein to a distal site^1^. How such interactions evolve is an unsolved problem in evolutionary biochemistry. It requires that both proteins (the regulator and the target) evolve a matching interface as well as some mechanism that translates binding of the regulator to a change at the active site of the target protein. If all residues that participate in this interface and the transmission mechanism have to evolve de novo, building such an interaction would require several substitutions in both proteins. Because long genetic trajectories involving several substitutions in multiple proteins are very unlikely to be fixed by random genetic drift, existing interactions are usually assumed to have been built up in incremental mutational steps. Each step would add a single interacting residue and would be driven to fixation by natural selection acting directly on a function associated with the interaction^2^. However, in a few protein systems, interfaces or allosteric pathways pre-existed fortuitously in one of the two partners^3–6^. This indicates that some aspects of these interactions arose by chance, which were then exploited by other components that arose later.

It remains unclear to what extent direct selection is necessary to fashion these remaining components of an interaction, such as the interaction surface of a new regulator that exploits a pre-existing surface on its target. In principle, these features could also be entirely accidental if they initially fixed for reasons unrelated to the interaction. In all well-studied cases we cannot answer this question because both components originated from within the same genome where the target and the regulator would have always encountered each other, so selection may or may not have acted to adapt the regulator to its new target^3–6^. Whether any biologically meaningful interaction ever truly arose by chance therefore remains unknown.

**Fig. 1:**
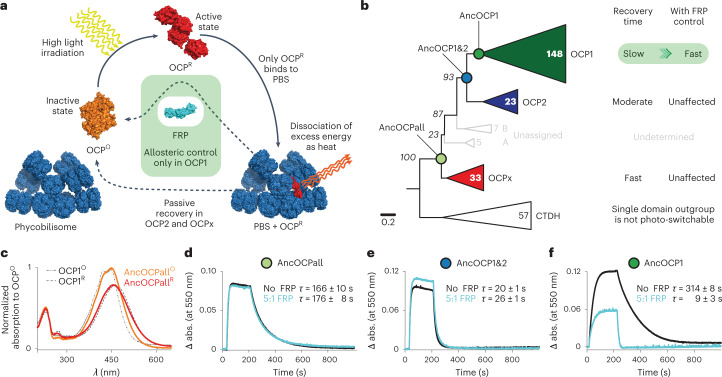
Evolution of allosteric control in OCP. **a**, Mechanism of cyanobacteria-exclusive, OCP-mediated photoprotection involving allosteric control by FRP (cyan) in OCP1 paralogues. Structures used (PDB IDs): 7EXT (ref. ^57^), 3MG1 (ref. ^58^), 4JDX (ref. ^25^) and 7SC9 (ref. ^29^). PBS, phycosbilisome. **b**, Reduced ML phylogeny of OCP paralogues with relative speed of recovery from photoconversion indicated, and reconstructed ancestral proteins (Anc) of selected clades. Cyanobacterial CTDHs are the outgroup. Bold numbers count taxa of designated OCP paralogues. Italic numbers are Felsenstein bootstrap probabilities of 100 replicates. Branch-lengths represent average substitutions per site. The complete tree is shown in Extended Data Fig. 1. **c**, Ultraviolet–visible absorption spectra of inactive orange and active red state of AncOCPall in comparison with extant OCP1 from *Synechocystis* sp. PCC 6803 (SYNY3; dashed lines). **d**–**f**, Recovery from photoconversion of ancestral OCPs at 20 °C with (cyan) or without SYNY3 FRP (black), and respective mean recovery time constants (*τ*) with s.d. of three independent replicates: AncOCPall (**d**), AncOCP1&2 (**e**) and AncOCP1 (**f**). Representative data sets are shown for clarity.

Here, we address this problem by studying the evolution of an allosteric interaction in the cyanobacterial photoprotection system^7,8^. Photoactive organisms must protect themselves from high light irradiation causing photodamage. In cyanobacteria, this protection is mediated by the orange carotenoid protein (OCP)^9,10^, a photoactive light intensity sensor with a carotenoid embedded symmetrically into its two domains that is able to switch conformation from an inactive orange (OCP^O^) to an activated red state (OCP^R^) under high light conditions^11^. Activated OCP^R^ binds to the cyanobacterial light-harvesting antenna complex, the phycobilisome, to dissipate excess phycobilisome excitation as heat^11,12^. Two OCP paralogues (OCP2 and OCPx) can detach from the phycobilisome and recover into OCP^O^ passively in the dark^11,13^. However, the most common paralogue OCP1 relies on an allosteric regulation for photo-recovery: OCP1 interacts with the fluorescence recovery protein (FRP), a small, dimeric regulator that terminates the interaction with the phycobilisome, and strongly accelerates the back-conversion of OCP^R^ into the resting orange state^14,15^ (Fig. 1a). Although the likely evolution of OCP from non-photo-switchable precursors has recently been demonstrated^16^, it is not yet known how FRP was recruited into the cyanobacterial photoprotection system as a new allosteric regulator.

## Results

### Ancestral OCPs are photo-switchable light intensity sensors

**Extended Data Fig. 1:**
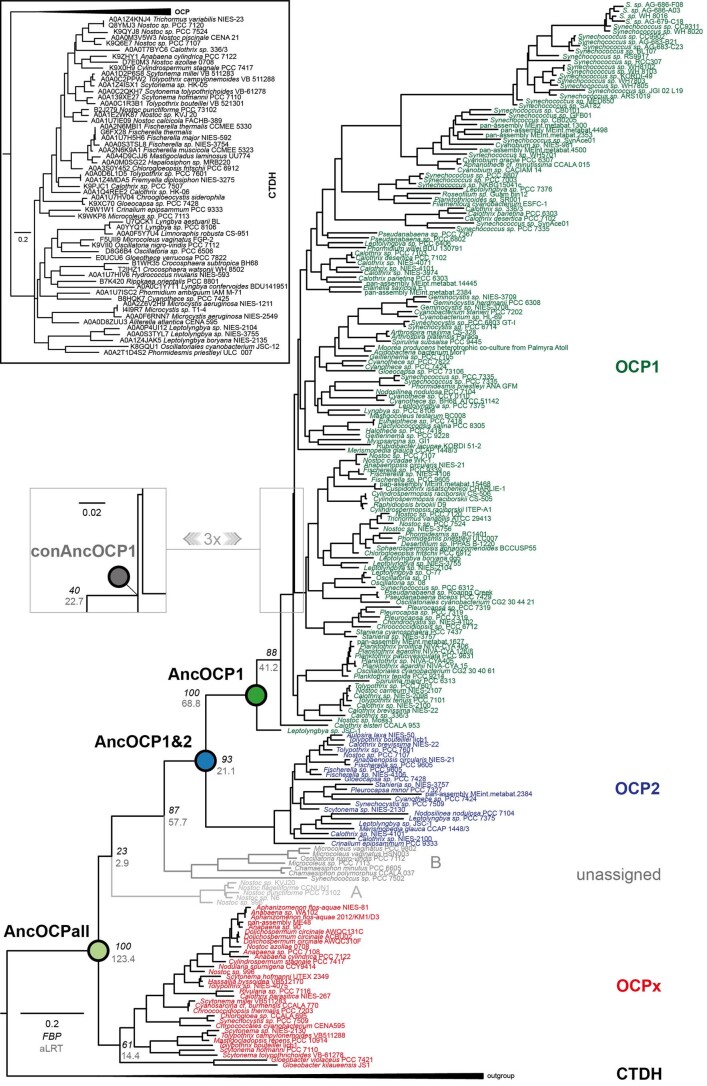
Complete phylogeny of OCP proteins. ML phylogeny of OCP proteins with reconstructed ancestral proteins (Anc) at labelled nodes, and cyanobacterial C-terminal domain-like proteins (CTDH) as the outgroup (insert with black outlines). OCP paralogs and ancestors are colour-coded as in Fig. 1b. We additionally tested a more conservative sequence for the last common ancestor of OCP1 (conAncOCP1, in grey) (Extended Data Fig. 3a+e, 4d,h,l,p,t) as well as alternative ‘altAll’ ancestors for every node on this tree (Extended Data Figs. 3a, 5a-l). Italic numbers are Felsenstein Bootstrap Probabilities (*FBP*) of 100 replicates. Grey numbers are approximate likelihood-ratio test values (aLRT). Branch-lengths represent average substitutions per site. Insert with grey outlines is a threefold zoom-in to properly display the branch topology in that area. Underlying multiple sequence alignment in Supplementary Data 1.

**Extended Data Fig. 2:**
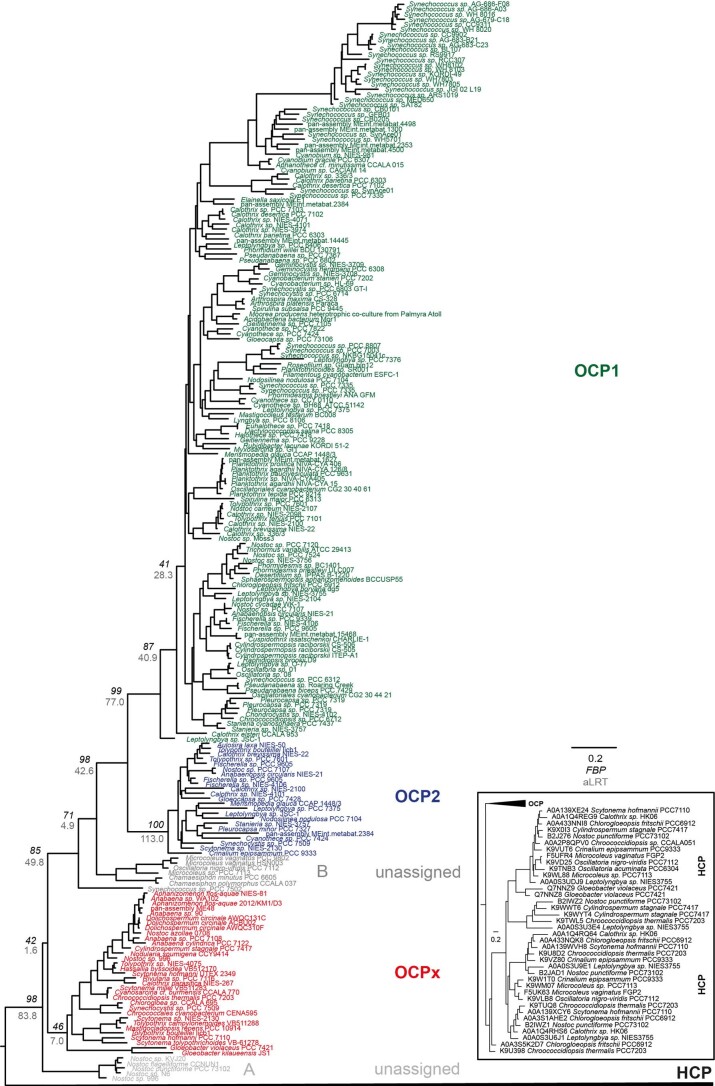
Alternatively rooted phylogeny of OCP proteins. ML phylogeny of OCP proteins like in Extended Data Fig. 1, but with cyanobacterial helical carotenoid proteins (HCP, insert) as the outgroup. Underlying multiple sequence alignment in Supplementary Data 1. No ancestors were reconstructed here.

To retrace the evolutionary origins of OCP1’s allosteric interaction with FRP, we first sought to understand how OCP paralogues evolved and when they gained the ability to be regulated by FRP. It has recently been shown that the first OCP probably evolved via a gene fusion event of two small proteins and that a linker addition provided photo-switchability^16^. Homologues of these single domain proteins can still be found in extant cyanobacteria, and have been termed helical carotenoid proteins (HCPs) and C-terminal domain-like homologues (CTDHs) that feature a common fold of nuclear transport factor 2 proteins (NTF2)^17^. We first inferred a maximum likelihood (ML) phylogeny of OCP proteins, using cyanobacterial CTDH sequences as the outgroup to root our tree (Fig. 1b and Extended Data Fig. 1). We further describe an alternative rooting using HCP sequences in Extended Data Fig. 2. Our phylogenetic tree is virtually identical to a recently published tree^16^, with OCPx, OCP2 and OCP1 each forming well-supported monophyletic groups. OCP1 and OCP2 are sister groups, to the exclusion of all other OCPs. Two more uncharacterized clades branch between the OCPx group and OCP1 and OCP2, which could be additional OCPx or represent separate paralogues.

**Extended Data Fig. 3:**
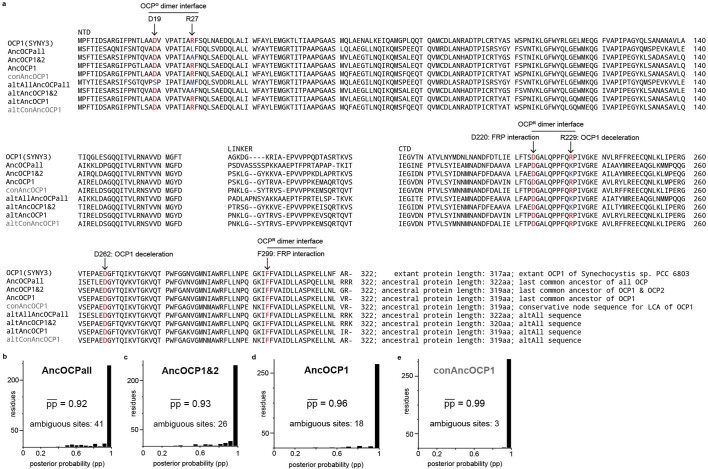
Reconstructed ancestral OCP sequences and their statistical robustness. **a**, Multiple sequence alignment of OCP1 from *Synechocystis* sp. PCC 6803 (SYNY3) with reconstructed ancestral OCP sequences and respective alternative sequences (alt). Important states for dimerization of OCP1^O^^16^, OCP1^R^^29^, deceleration of OCP1, and interaction with FRP^7^ are indicated, and red if conserved or blue if not. Numeration of residues follows SYNY3 OCP1. C-terminal domain (CTD), linker, and N-terminal domain (NTD) regions are labelled accordingly. The more conservative ancestral OCP1 (conAncOCP1) and its alternative sequence that do not appear in the main text are greyed. **b-e**, Distribution of posterior probabilities (pp) per site with 20 bin categories per reconstructed sequence with the mean and the number of ambiguous sites shown. Sites were considered ambiguous if pp > 0.2 for the state with the second highest pp and were replaced with those states in the alt ancestors.

**Extended Data Fig. 4:**
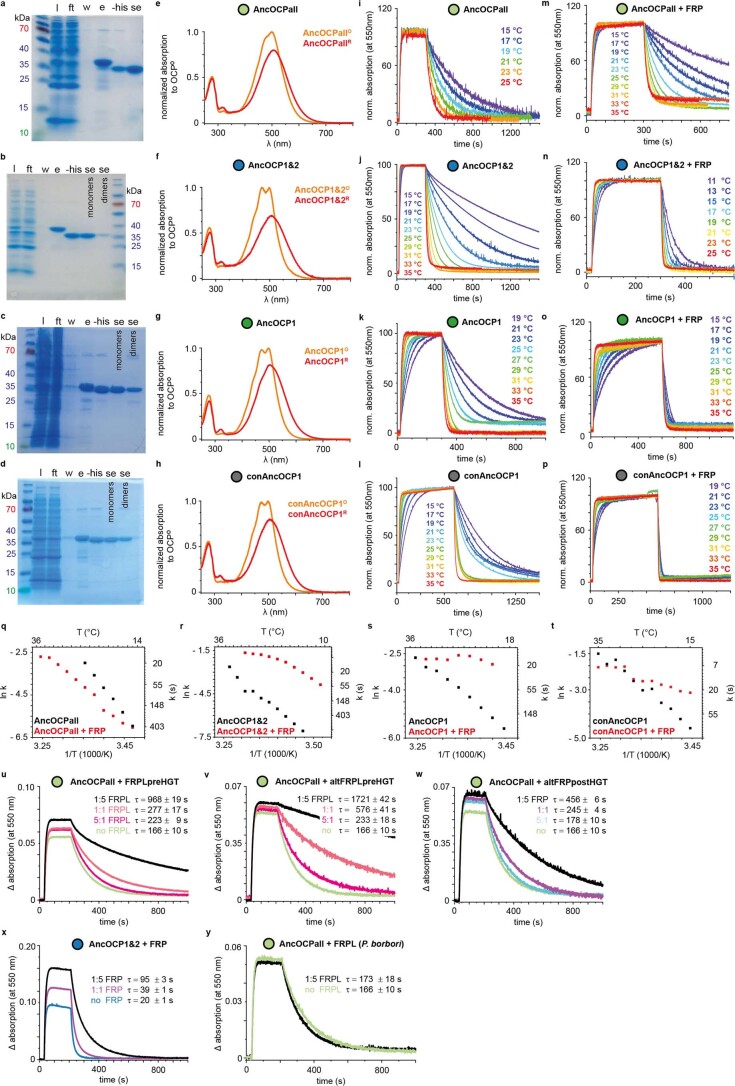
Biochemistry of ancestral OCPs. **a-d**, 12 % SDS polyacrylamide gels of ancestral protein purifications. l, lysate. ft, flow through. w, wash. e, elution. -his, after his-tag cleavage. se, after size exclusion chromatography. Purifications were repeated three times with similar results. **e-h**, UV-Vis absorption spectra of inactive orange and active red state of ancestral OCPs. **i-p**, Recovery from photoconversion of ancestral OCPs with (in molar ratios of 5 OCP to 1 FRP) or without extant FRP from *Synechocystis* sp. PCC 6803 (SYNY3) as indicated at different temperatures. **q-t**, Arrhenius plots of recovery from photoconversion with (red) or without SYNY3 FRP (black). **u-y**, Recovery from photoconversion of ancestral OCPs either alone or with different ancestral FRPs or ancestral FRPLs or extant FRPL from *Pseudomonas borbori* in different molar ratios as indicated at 20 °C with respective mean recovery time constants (τ) and s.d. of three independent replicates. Representative data sets are shown for clarity.

We used ancestral sequence reconstruction to infer the amino acid sequences of ancestral OCPs at the internal nodes of our tree and along the lineage towards FRP-regulated OCP1. We focused on three proteins from the last common ancestor (LCA) of all extant OCP (AncOCPall) to the LCA of OCP1 and OCP2 paralogues (AncOCP1&2) up to the LCA of extant OCP1 (AncOCP1), which were reconstructed with average posterior probabilities across sites between 0.92 and 0.96 (Fig. 1b and Extended Data Fig. 3a–e). We resurrected these ancestral OCP proteins heterologously in *Escherichia coli*, and purified them for in vitro characterization. All ancestral OCPs are photo-switchable light intensity sensors with a bound echinenone as the favoured carotenoid (Fig. 1c and Extended Data Fig. 4a–h). AncOCPall shows a moderate time constant for the OCP^R^ to OCP^O^ back-conversion of 166 ± 10 s (similar to extant OCP2, ref. ^16^). The recovery constant decreases to 20 ± 1 s in AncOCP1&2 (faster than extant OCPs), but drastically increases in AncOCP1 to 314 ± 8 s (as in extant OCP1) (Fig. 1d–f and Extended Data Fig. 4i–l). Our data show that slow photo-recovery is a feature that evolved along the branch to OCP1, consistent with the theory that only OCP1 paralogues require FRP for allosterically accelerated recovery.

### FRP-accelerated recovery evolved along the branch to OCP1

We next tested the effect of an extant FRP from *Synechocystis* sp. PCC 6803 on the recovery times of our ancestral OCPs. The two earlier ancestors are unaffected by FRP, whereas AncOCP1 is only able to rapidly recover in the presence of FRP (in molar ratios of five OCP to one FRP), which accelerates the OCP^R^ to OCP^O^ back-conversion by about 97% (similar to extant OCP1) (Fig. 1d–f and Extended Data Fig. 4m–t). As AncOCP1&2 is unaffected by FRP, the allosteric acceleration of OCP’s recovery evolved after the gene duplication event that gave rise to OCP1 and OCP2 paralogues, only along the branch to OCP1.

**Extended Data Fig. 5:**
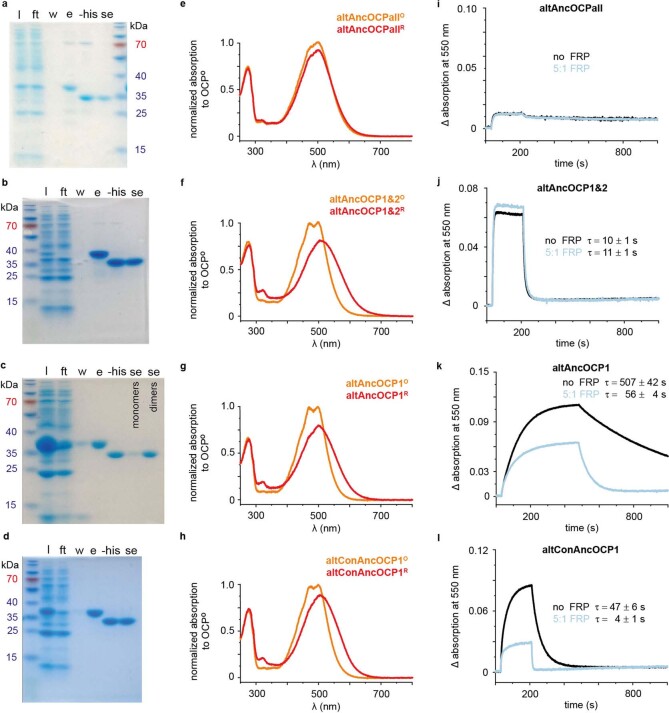
Biochemistry of alternative ancestral OCPs. **a-d**, 12 % SDS polyacrylamide gels of alternative ancestral protein purifications. l, lysate. ft, flow through. w, wash. e, elution. -his, after his-tag cleavage. se, after size exclusion chromatography. Purifications were repeated three times with similar results. **e-h**, UV-Vis absorption spectra of inactive orange and active red state of alternative ancestral OCPs. **i-l**, Recovery from photoconversion of alternative ancestral OCPs with (cyan) or without (black) extant FRP from *Synechocystis* sp. PCC 6803 at 20 **°**C with respective mean recovery time constants (τ) and s.d. of three independent replicates. Representative data sets are shown for clarity; altAncOCPall is barely photo-switchable.

We tested the robustness of our conclusions to statistical uncertainties in our resurrected sequences by additionally resurrecting one less likely, but still statistically plausible, alternative sequence per ancestor (see Methods for details). Biophysical characterizations of these alternative ancestral OCP proteins confirm that slow recovery and acceleration by FRP evolved along the branch leading to OCP1 (Extended Data Fig. 5a–l).

### FRP was acquired laterally early in cyanobacterial history

**Extended Data Fig. 6:**
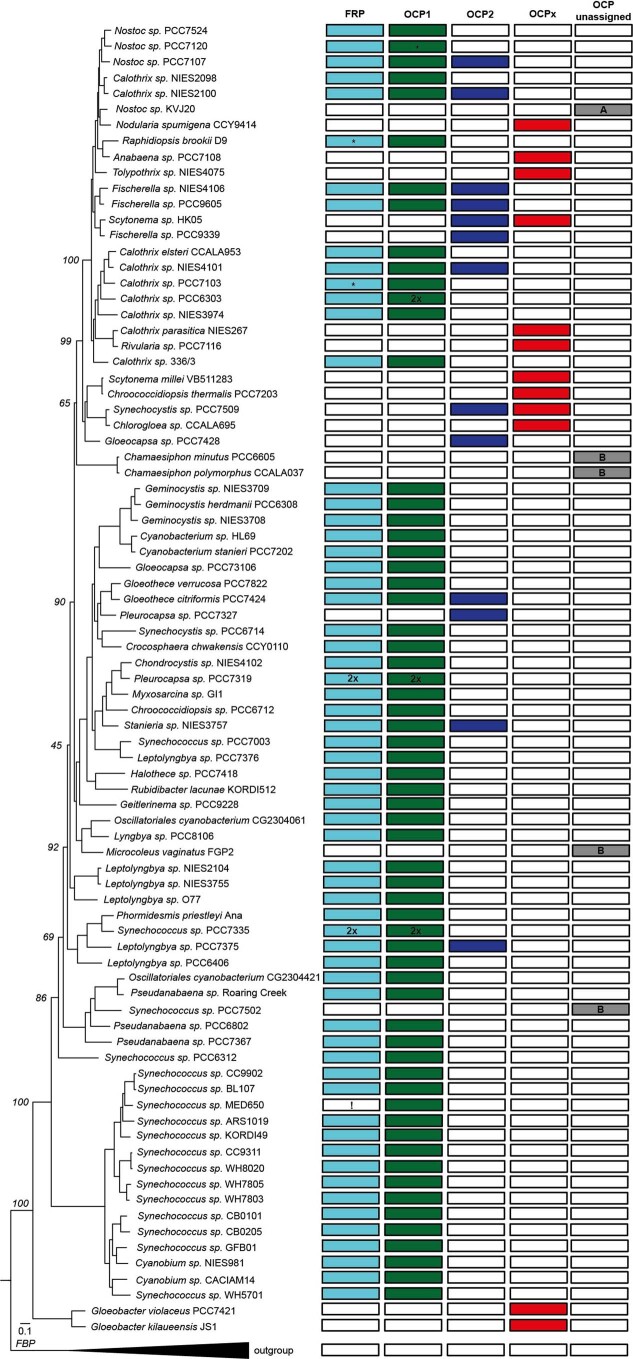
Species phylogeny of OCP-containing cyanobacteria. ML species phylogeny with Felsenstein Bootstrap Probabilities (*FBP*) of 100 replicates in italics. The appearance of FRP and OCP paralogs are mapped next to the phylogeny. Asterisks indicate multispecies entries in the BLAST database^39^. The exclamation point marks the only strain lacking FRP while having OCP1. Underlying amino acid sequence alignment in Supplementary Data 1.

We next asked when FRP first appeared in cyanobacterial genomes, relative to the gene duplication that produced FRP-regulated OCP1. To answer this, we inferred a ML species phylogeny of OCP-containing cyanobacterial strains found on our OCP tree and mapped the presence of FRP and OCP paralogues onto it (Extended Data Fig. 6). Virtually all OCP1-containing genomes also contain FRP, suggesting FRP was gained close in time to the duplication that produced OCP1. Exactly where on the species phylogeny the successive OCP duplications occurred is difficult to tell, because OCP2 and OCPx paralogues have very sporadic distributions, and the relationships within each OCP clade are only poorly resolved. Gloeobacteria, which on our and others’ species phylogenies^18–21^ are sister to all other cyanobacteria, only possess OCPx, whereas groups branching immediately after already have OCP1 and FRP or OCP2 or both. This suggests that the duplication that produced OCP1 and OCP2 happened relatively quickly after *Gloeobacter* spp. split off from all other cyanobacteria, and that FRP was recruited into the system around the same time.

**Fig. 2:**
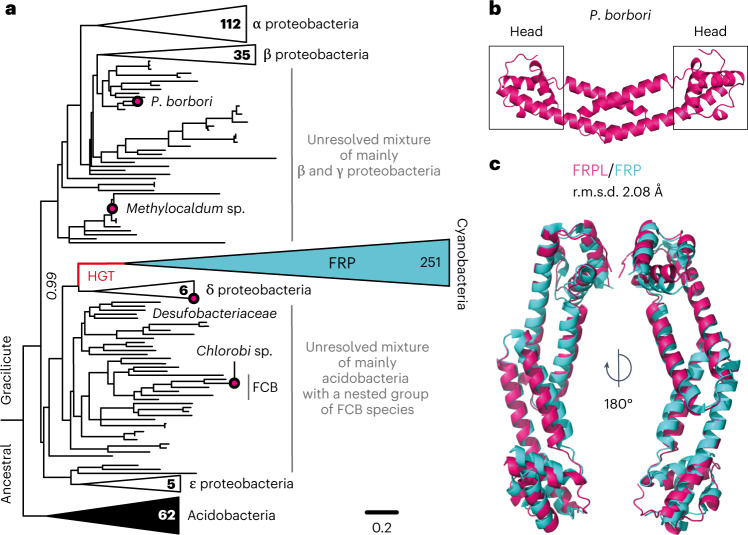
FRP evolved from structurally highly similar proteins through horizontal transfer. **a**, Reduced ML phylogeny of cyanobacterial FRP (cyan), and homologous FRPL proteins with examined ones in this study indicated by a magenta circle and their host species’ name. Bold numbers count taxa of collapsed bacterial groups. Italic number indicates TBE of 100 replicates. The tree was rooted between proteobacteria and acidobacteria, and indicates a HGT between delta-proteobacteria and cyanobacteria (red line). Branch lengths represent average substitutions per site. The complete tree is shown in Supplementary Fig. 1. **b**, Crystal structure of the FRPL homo-dimer from *P. borbori* at 1.8 Å with head domains indicated (PDB ID 8AG8) **c**, Rotated overlay with FRP (PDB ID 4JDX from *Synechocystis* sp. PCC 6803, ref. ^25^). r.m.s.d., root-mean-square deviation.

Our next goal was to understand the origin of FRP. Homologues of FRP (termed FRP-like, FRPL) can also be found in distantly related bacteria^8,22^, mainly proteobacteria and acidobacteria, suggesting an origin far beyond cyanobacteria. To test this theory, we extensively searched for FRP homologues in and outside cyanobacteria and inferred a ML phylogeny. Our tree features a highly supported split between all FRPs and all FRPLs (Fig. 2a). A small group of delta-proteobacterial FRPLs branches closest to the cyanobacterial FRP group with high statistical support (approximate likelihood-ratio test (aLRT) = 60.9, transfer bootstrap expectations (TBE) of 0.99). However, in some bootstrap runs FRPLs of other bacterial taxa with long terminal branches jump into this group, resulting in poor Felsenstein bootstrap support (FBP = 0.51), but the delta-proteobacterial FRPLs remain sister to FRP in all runs. Further FRPLs are sporadically distributed in the proteobacteria and acidobacteria, and mostly found in uncultured species (and entirely absent in model organisms). Within different groups of proteobacteria our tree becomes poorly resolved, probably owing to the short length of FRP and FRPL proteins.

We rooted the tree between acidobacteria and proteobacteria within the FRPL group as the most parsimonious root hypothesis. This root indicates a horizontal gene transfer (HGT) from an ancestral delta-proteobacterium into an ancestral cyanobacterium, and further indicates many sporadic losses of FRPL in acidobacteria and proteobacteria (Fig. 2a). A root within the FRP group would in contrast require more and less plausible HGT events: at least from cyanobacteria into only a small set of proteobacteria, then into acidobacteria and then from relatively modern acidobacteria into early proteobacteria. A root between FRPs and FRPLs would require an origin of the protein in the LCA of all bacteria^23^, which would indicate losses in many large bacterial groups as well as the same temporally implausible transfer from modern acidobacteria into the LCA of proteobacteria (see Supplementary Discussion for details). As a consequence, our results indicate that FRP was most probably horizontally acquired by an ancestral cyanobacterium early in cyanobacterial history.

### FRP evolved from structurally highly similar proteins

**Extended Data Fig. 7:**
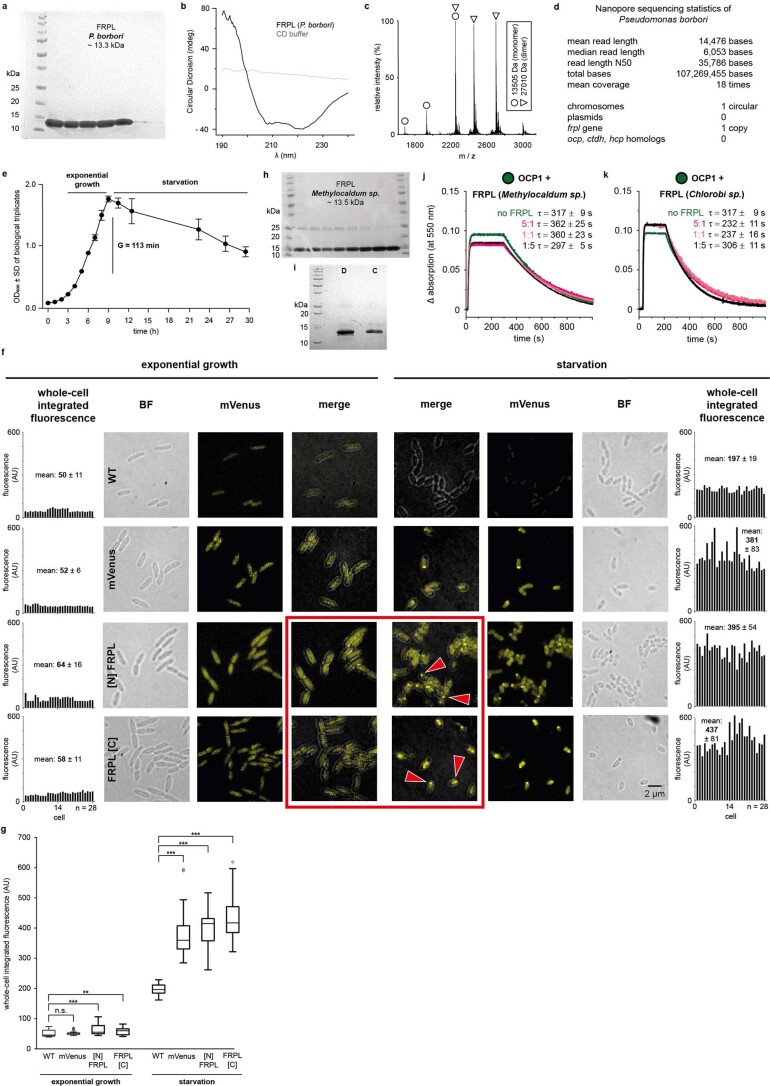
Characterization of extant FRPLs. **a, h** + **i**, 15 % SDS polyacrylamide gels of *P. borbori*, *Methylocaldum* sp.*, Desulfobacteriaceae* (D), and *Chlorobi* sp. (C) FRPL after size exclusion chromatography. Purifications were repeated three times with similar results. **b**, Circular dichroism (CD) spectra of *P. borbori* FRPL (black) in CD buffer (grey). **c**, Native mass spectrometry data of *P. borbori* FRPL. **d**, Nanopore sequencing statistics. **e**, Growth curve of *P. borbori* in biological triplicates with means and standard deviation (SD) shown, and determination of the generation time (G) during exponential growth. **f**, Epi-fluorescence microscopy of *P. borbori* strains expressing either none (WT), mVenus only (mVenus), or FRPL fusion proteins with either N- or C-terminal mVenus fusion. Whole-cell integrated fluorescence with the mean and s.d., the brightfield (BF) image, the GFP channel signal (mVenus), and an overlay of both (merge) is shown. Red arrows point to signal foci at the cell poles. Scale bar represents 2 μm and is applicable for all images. **g**, Two-sided Welch’s t-tests were performed to compare mean whole-cell integrated fluorescence with *** p < 0.001, ** p = 0.013, n.s., not significant (p = 0.580); n = 28 cells per condition. Boxes extend from lower to upper interquartile values of the data, with a line at the median. Whiskers display data within ± 1.5 interquartile ranges. Circles are outliers. **j** + **k**, Recovery from photoconversion of OCP1 from *Synechocystis* sp. PCC 6803 with extant FRPL as indicated at 20 °C with respective mean recovery time constants (τ) and SD of three independent replicates. Representative data sets are shown for clarity.

To understand the ancestral state of FRPL proteins before they were transferred into cyanobacteria, we heterologously expressed, purified and characterized the FRPL from one of the few isolated, mesophilic bacteria that feature FRPL (PbFRPL): the gamma-proteobacterium *Pseudomonas borbori*, a close relative of *P. aeruginosa*^24^. Circular dichroism spectroscopy of PbFRPL showed the typical all alpha-helical fold, previously found in FRP in solution, and native mass spectrometry confirmed the distinctive dimeric state^8,14^ (Extended Data Fig. 7a–c). We solved PbFRPL’s crystal structure to a resolution of 1.8 Å (Table 1). The N-terminal domain consists of two antiparallel alpha-helices of about 50 Å in length and features a homo-dimerization interface similar to those in FRPs with an estimated buried surface of around 675 Å^2^. The C-terminal head domain, that in FRP is thought to interact with OCP1 (refs. ^25–27^), is also present in PbFRPL, and constitutes three interlocking alpha-helices. Overall, PbFRPL and FRP from *Synechocystis* sp. PCC 6803 (Protein Data Bank (PDB) ID 4JDX, ref. ^25^) superpose with a root-mean-square deviation of 2.08 Å (Fig. 2b,c). PbFRPL’s structural properties are therefore extremely similar to those of cyanobacterial FRP.

**Table 1:** Crystallographic data collection and refinement statistics

	PbFRPL
**Data collection**	
Space group	*P*4_3_ 2_1_ 2
Cell dimensions	
*a*, *b*, *c* (Å)	53.46, 53.46, 92.67
α, β, γ (°)	90, 90, 90
Resolution (Å)	46.334–1.8 (1.864–1.8)
*R*_merge_	0.05548 (0.5827)
*I* / σ*I*	32.02 (2.99)
Completeness (%)	98.21 (97.13)
Redundancy	22.2 (12.9)
CC_1/2_	1 (0.96)
**Refinement**	
Resolution (Å)	35–1.8 (1.864–1.8)
No. reflections	12,829 (1,218)
*R*_work_ / *R*_free_	0.2271 (0.2755) / 0.2322 (0.3964)
No. atoms	
Protein	877
Ligand / ion	13
Water	73
*B*-factors	
Protein	37.94
Ligand / ion	46.87
Water	43.55
Ramachandran (%)	
Favoured	100
Allowed	0
Outliers	0
Root-mean-square deviations	
Bond lengths (Å)	0.02
Bond angles (°)	1.45

Values in parentheses are for highest-resolution shell.

It is unclear what function FRPLs carry out, but it cannot be regulating OCP because genomes containing FRPL contain neither OCPs nor homologues of their N-terminal domain- or CTD-like proteins (HCP and CTDH, respectively). In *P. borbori*, the *frpl* gene is encoded on its single chromosome, and we did not find any OCP, HCP or CTDH homologues (Extended Data Fig. 7d). Epi-fluorescence microscopy of PbFRPL fused to an mVenus fluorophore and expressed from a plasmid under its native promotor in *P. borbori* showed a homogeneous distribution across the whole cell during exponential growth and an additional concentration at the cell poles upon starvation with increased whole-cell integrated fluorescence by about 2.5- to 3.4-fold above wild-type increase (Extended Data Fig. 7e–g). Keeping in mind that we cannot control for protein copy number here, it is noticeable that PbFRPL localization and quantity change in response to starvation. Our data indicate that despite their extremely similar structures, FRPLs carry out a potentially stress-related function that must be totally unrelated to OCPs and the regulation of photoprotection.

### FRPL evolved the ability to interact with OCP by chance

**Fig. 3:**
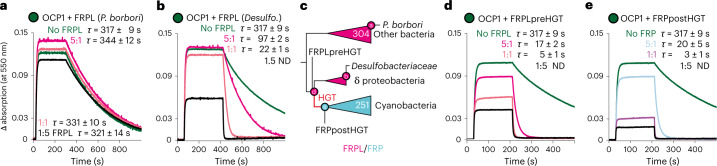
Some FRPLs could fortuitously accelerate OCP’s recovery from photoconversion before they were transferred into cyanobacteria. **a**,**b**, Recovery from photoconversion of extant OCP1 from *Synechocystis* sp. PCC 6803 (SYNY3) with extant FRPL of *P. borbori* (**a**) or a *Desulfobacteriaceae (Desulfo.)* species (**b**) at different molar ratios as indicated at 20 °C with respective mean recovery time constants (*τ*) and s.d. of three independent replicates. Representative data sets are shown for clarity. ND, not determinable. **c**, Schematic FRP(L) phylogeny with reconstructed ancestral proteins, and extant FRPLs tested. The complete tree is shown in Supplementary Fig. 1. **d**,**e**, Recovery from photoconversion of extant SYNY3 OCP1 with ancestral FRPL (FRPLpreHGT) that existed before (**d**), and ancestral FRP (FRPpostHGT) that existed after the HGT (**e**) at different molar ratios as indicated at 20 °C with respective mean recovery time constants (*τ*) and s.d. of three independent replicates. Representative data sets are shown for clarity.

**Extended Data Fig. 8:**
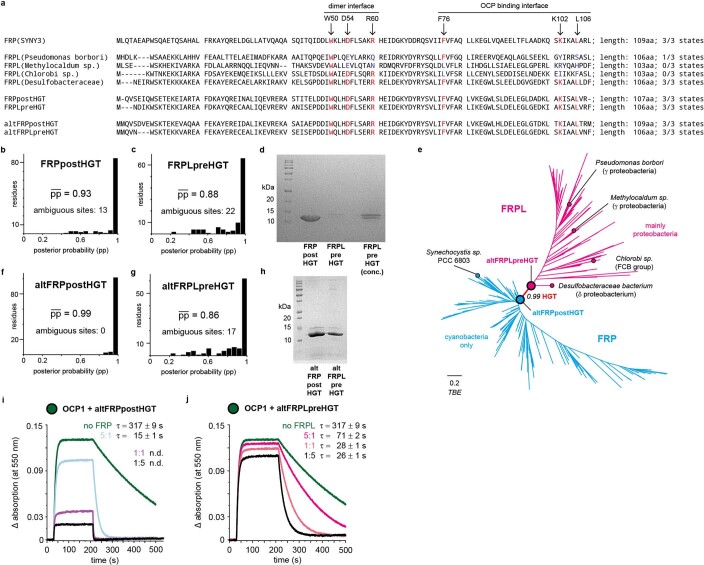
Resurrected ancestral FRP and ancestral FRPL sequences and their statistical robustness. **a**, Amino acid sequence alignment of FRP from *Synechocystis* sp. PCC 6803 (SYNY3) with extant and reconstructed ancestral FRPLs and ancestral FRPs. Important sites for homo-dimerization and interaction with OCP1 in FRP are pointed out^7,8^, and red if conserved or blue if not. Numeration follows SYNY3 FRP. ML trees for the reconstructions in Supplementary Fig. 1 + 2. **b** + **c**, **f** + **g**, Distribution of posterior probabilities (pp) per site with 20 bin categories per reconstructed sequence with the mean and the number of ambiguous sites with pp > 0.2 for the state with the second highest pp shown. **d** + **h**, 15 % SDS polyacrylamide gels of ancestral proteins after size exclusion chromatography. Purifications were repeated three times with similar results. conc., concentrated. **e**, Unrooted initial FRP(L) phylogenetic tree used for reconstruction of alternative (alt) ancestors at indicated nodes. Branch-lengths represent average substitutions per site. Full tree in Supplementary Fig. 2. HGT, horizontal gene transfer. TBE, Transfer Bootstrap Expectation. **i** + **j**, Recovery from photoconversion of SYNY3 OCP1 with alternative ancestral FRP (altFRPpostHGT) or alternative ancestral FRPL (altFRPLpreHGT) as indicated at different molar ratios at 20 °C with respective mean recovery time constants (τ) and s.d. of three independent replicates. Representative data sets are shown for clarity. n.d., not determinable.

The shared fold of FRPL and FRP suggests FRPLs may be able to interact productively with OCP, meaning that they may have needed no additional modifications after being transferred into cyanobacteria to immediately function in their photoprotection system. To test this, we purified several FRPLs from extant species, and examined their effect on extant OCP1’s photo-recovery. We chose FRPLs from four organisms that span the diversity of FRPL-containing bacterial groups on our phylogenetic tree: *P. borbori*, *Methylocaldum* sp. (another gamma-proteobacterium), *Chlorobi* sp. (an FCB group species) and a delta-proteobacterium of the *Desulfobacteraceae* family, which represents one of the closest extant sequences to the HGT event into cyanobacteria on our tree (Fig. 2a). FRPL from *P. borbori*, *Methylocaldum* sp. and *Chlorobi* sp. had virtually no effect on OCP1’s photo-recovery. However, the *Desulfobacteraceae* FRPL showed the typical acceleration of OCP1’s recovery from photoconversion by about 93% (when incubated in an equimolar ratio of OCP1 to FRPL), compared to OCP1 alone (Fig. 3a,b and Extended Data Fig. 7h–k). This indicates that the ability to regulate OCP1 already existed at the moment of the HGT event that first transferred FRP into cyanobacteria. To further test this theory, we additionally resurrected two ancestral proteins: FRPLpreHGT that is the latest FRPL we can reconstruct before the HGT event and FRPpostHGT that represents the LCA of all FRP in cyanobacteria after the HGT (Fig. 3c). Both ancestral proteins also show the typical accelerating FRP effect on OCP1’s photo-recovery, performing almost as well as extant FRP (Fig. 3d,e and Extended Data Fig. 8a–d). This inference is further robust to alternative ancestral FRP and ancestral FRPL proteins with slightly different sequences that, on the basis of an initial FRP(L) phylogeny we had inferred earlier with fewer sequences in total (Extended Data Fig. 8e–j).

Taken together, our results show that most FRPLs cannot function as allosteric regulators of OCP1, but that a small subgroup of them fortuitously acquired this ability. Because this happened in a genome that contained no OCP, this ability is entirely accidental and cannot have been the result of direct natural selection. In principle, this would have allowed the protein to function in the totally unrelated photoprotection system of cyanobacteria the moment it was first transferred into their genomes.

### The OCP–FRP interface predates the allosteric effect

**Fig. 4:**
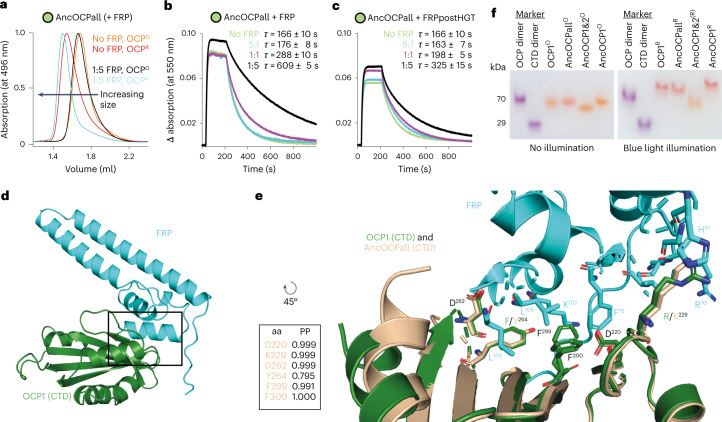
Ancestral OCPs could interact with FRP through a conserved dimer interface before FRP was acquired. **a**, Analytical SEC of AncOCPall and AncOCPall–FRP complexes with (OCP^R^) or without constant blue light illumination (OCP^O^) during chromatography. **b**,**c**, Recovery from photoconversion of AncOCPall with different molar ratios of extant FRP from *Synechocystis* sp. PCC 6803 (SYNY3) (**b**) or FRPpostHGT (**c**) at 20 °C with respective mean recovery time constants (*τ*) and s.d. of three independent replicates. Representative data sets are shown for clarity. Data for ‘no FRP’ and ‘5:1 FRP’ in **b** are taken from Fig. 1d for comparison. **d**, AlphaFold2 model of the interaction between FRP (cyan) and the CTD of SYNY3 OCP1 (green). **e**, Rotated zoom (of black framed area in **d)** into the binding interface, with AncOCPall (in wheat) overlaid onto OCP1. Amino acids involved in binding are labelled. Sites conserved in both OCPs are in black. Nitrogen in blue and oxygen in red. Residue numbers follow SYNY3 OCP1. The insert shows the PP for indicated amino acids in the binding interface of the reconstructed AncOCPall protein. **f**, Native PAGE of ancestral OCP^O^ without illumination (left), and OCP^R^ during constant blue light illumination (right) show their oligomeric states. Comparison with OCP1 (refs. ^29,58^) indicates conserved dimerization interfaces that differ between OCP^O^ and OCP^R^. An OCP mutant (70 kDa) and the CTD of OCP1 (29 kDa) that both form illumination-independent dimers were used as molecular markers. Experiments were repeated three times with similar results.

Since some FRPLs seem primed for the interaction with OCP even before they came into cyanobacteria, we reasoned that the interface for their interaction may also already be present in AncOCPall, even if the allosteric connection to accelerate the photo-recovery had not yet fully evolved. Analytical size-exclusion chromatography (SEC) of photoactivated, red forms of AncOCPall (AncOCPall^R^) incubated with extant FRP showed increased size relative to AncOCPall^R^ alone (Fig. 4a), indicating that FRP already binds to AncOCPall^R^. We asked whether we could trigger the allosteric response by adding FRP in excess to the OCP^R^toOCP^O^ recovery reaction, and repeated our initial experiments (Fig. 1d), but this time using a much larger molar ratio of FRP relative to OCP. To our surprise, instead of an acceleration, the recovery time drastically increased from 166 ± 10 to 288 ± 10 s and 609 ± 5 s, using an equimolar amount (of OCP to FRP) and a fivefold molar excess of FRP, respectively (Fig. 4b). This deceleration also appeared in AncOCP1&2, and if adding any of the ancestral FRPs or ancestral FRPLs (Fig. 4c and Extended Data Fig. 4u–x). To rule out that this slowing down is only caused by steric effects or molecular crowding, we repeated the experiments with PbFRPL (which has virtually no effect on OCP1’s recovery time, even if added in molar excess: Fig. 3a), and likewise found virtually no effect on AncOCPall’s recovery (Extended Data Fig. 4y).

Binding FRP alone is thus not sufficient for the accelerating allosteric effect to happen. Instead, it impedes photo-recovery of AncOCPall at high molar excess of FRP. Repetitive weak binding or an FRP that does not dissociate on the right timescale could interrupt or delay the recovery process of AncOCPall. Further, structural features on the OCP side such as the flexible linker loop between the N-terminal domain and CTD or the short N-terminal extension may need to be further fine-tuned for the complex and highly efficient allosteric response of extant OCP1 to take place^16,26^.

Our experiments show that the LCA of all OCPs already had a latent ability to interact with FRP, although this interaction was not yet capable of accelerating recovery. This implies that at least this interaction potential between OCP and FRP evolved purely by chance, even before these proteins first encountered each other in an ancestral cyanobacterium.

**Extended Data Fig. 9:**
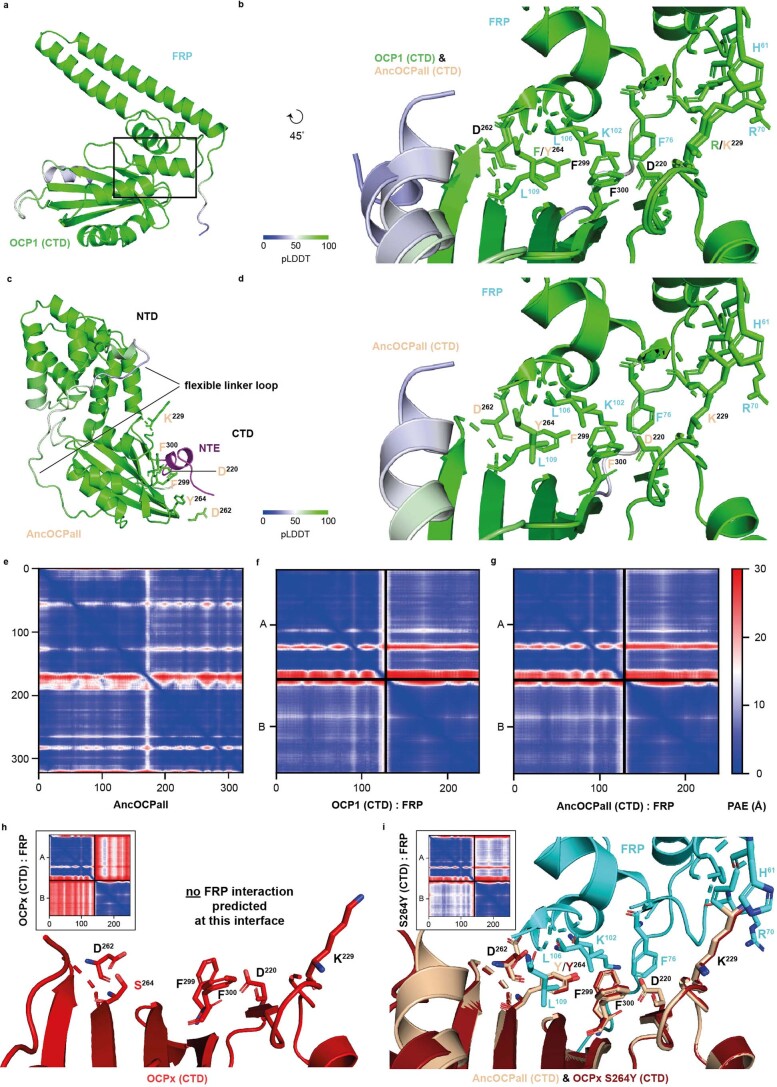
The OCP-FRP interaction is predicted with high confidence. **a** + **b**, Per-residue estimate of confidence (pLDDT) of AlphaFold2 models shown in Fig. 4d+e. **c**, Confidence of the predicted full-length AncOCPall with indicated residues in the C-terminal domain (CTD) involved in the predicted interaction with FRP from *Synechocystis* sp. PCC 6803 (SYNY3) that are blocked by the N-terminal extension (NTE, in magenta) in the compact, orange state of AncOCPall predicted here. NTD, N-terminal domain. **d**, Confidence of the modelled interaction between AncOCPall and SYNY3 FRP. **e-g**, Predicted aligned errors (PAE). **h** + **i**, AlphaFold2 models of OCPx’s CTD from *Gloeobacter kilaueensis* JS1 do not predict an interaction with SYNY3 FRP at the expected interface (consistent with experimental data^30^), unless serine (S) at position 264 (SYNY3 numeration) is changed for tyrosine (Y), the ancestral state in AncOCPall that is further shown in overlay here. Inserts show PAEs.

To understand the structural basis of this latent affinity, we inferred an AlphaFold2 (ref. ^28^) model of the OCP1–FRP complex. It confidently predicted an interaction between the CTD of OCP1 and FRP (Fig. 4d and Extended Data Fig. 9a,f) that is consistent with previous small-angle X-ray scattering data^27^. The interaction exploits the same hydrophobic surface as OCP1 uses to dimerize in its red state on the phycobilisome^29^. FRP has been theorized to favour detachment of OCP1^R^ from the phycobilisome by down-shifting the association constant of binding and accelerating recovery by competing with this dimer interface in OCP1 (ref. ^27^). The residues and charges shown to be important for this dimer interface are also present in our ancestral OCPs (Extended Data Fig. 3a), potentially explaining why FRP can already interact with AncOCPall. We tested this hypothesis in two ways: first, we inferred an AlphaFold2 model of the CTD of AncOCPall, and compared its surfaces to OCP1’s CTD. AncOCPall possesses the same hydrophobic surface as OCP1 with virtually all interface sites or charges identical between the two proteins. AlphaFold2 additionally predicts an interaction between this surface in AncOCPall and FRP (Fig. 4e and Extended Data Fig. 9b–e,g). Second, this model further indicates that dimerization in the red state should be an ancestral feature of all OCPs. To test this, we used Native PAGE to understand whether our ancestral OCPs also dimerize in their activated, red form. Consistent with our prediction, activation leads to the formation of complexes consistent in size with homo-dimers in AncOCPall^R^ and AncOCP1^R^. We did not detect red dimers in AncOCP1&2, probably due to its extremely rapid recovery time that technically impedes sustaining the red form in the gel (Fig. 4f).

Together, this indicates that the binding surface exploited by FRP is an ancient dimer interface of the red form of OCP that was already present in the LCA of all OCPs, even before FRP was recruited into the cyanobacterial system.

### OCP and FRPL drifted in and out of their ability to interact

OCPx paralogues are not affected by FRP any more^16,30^. To identify the underlying structural changes between AncOCPall and OCPx, we repeated the interaction predictions with the CTD of an extant OCPx from *Gloeobacter kilaueensis* JS1. AlphaFold2 did not predict the interaction interface between FRP and this OCPx unless we changed a conserved serine in the potential interface back to the ancestral tyrosine of AncOCPall (Extended Data Fig. 9h,i). This suggests that OCP proteins drifted in and out of the structural state that enables interaction with FRP.

To understand the structural causes of why only some FRPLs accelerate OCP1’s recovery from photoconversion, we finally compared the sequences of different FRPLs. In our AlphaFold2 model, phenylalanine 76, lysine 102 and leucine 106 in FRP of *Synechocystis* sp. PCC 6803 are in contact with OCP1. Most FRPLs do not have all three states together, but occasionally have one or two of these states. *P. borbori* FRPL for instance has the phenylalanine, but features a tyrosine at position 102 and a serine at position 106 (Extended Data Fig. 8a). Other FRPLs have the lysine, but lack the phenylalanine or the leucine. This shows that the important states for the interaction with OCP1 individually come and go across the FRPL phylogeny. All three states only appeared together in FRPLs along the linage towards delta-proteobacteria and cyanobacteria. It is remarkable that the HGT into cyanobacteria happened exactly in this narrow window of full compatibility.

## Discussion

**Fig. 5:**
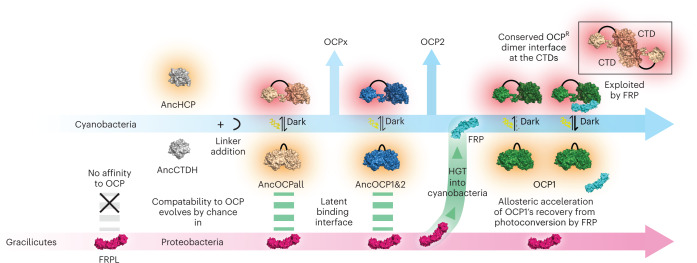
Evolutionary origin of the allosteric regulator FRP in the cyanobacterial OCP-mediated photoprotection system. The first photo-switchable OCP that undergoes conformational change from a closed orange to an open red state on high light irradiation was formed in a fusion event of an ancestral HCP (AncHCP) and an ancestral CTD-like homologue (AncCTDH) via a linker addition^16^. An FRP-like protein (FRPL) was horizontally transferred (HGT) into the unrelated cyanobacterial system after a latent binding interface for ancestral OCPs had already evolved by chance. FRP now exploits the conserved CTD dimerization interface of OCP^R^ to strongly accelerate OCP1’s recovery from photoconversion. OCP structure used here for illustration only is PDB ID 3MG1 (ref. ^58^).

Here, we have reconstructed the evolution of an allosteric interaction in the cyanobacterial photoprotection system. Together with previous work on the initial evolution of OCP^13,16^, the picture that emerges is a remarkable example of evolutionary tinkering:^31^ OCPs were most likely created by a gene fusion event that required nothing but a flexible linker to create a photo-switchable protein out of two non-switchable components^16^. Horizontal acquisition of FRP then introduced a new component that could allosterically accelerate ground state recovery in OCP1 without any further modification. Creating the fully functional OCP1–FRP system then only required substitutions in OCP that converted an initially unproductive interaction with the CTD into one that results in an acceleration of photo-recovery (Fig. 5). Because we cannot time the acquisition of FRP precisely relative to our OCP ancestors, we do not know whether these substitutions occurred before or after FRP was acquired. If they had happened before, the regulatory interaction between OCP1 and FRP would have been completely functional the moment FRP was horizontally acquired. Another known function of FRP is the facilitation of OCP1 detachment from the phycobilisome by shifting the OCP^R^–phycobilisome binding equilibrium constant^15^. Although this aspect was not surveyed in our study, we imagine that competitive FRP binding to an ancestral OCP^R^ dimer could also facilitate the detachment from the phycobilisome or at least impede binding to it, in effect generating a potential ancestral mode of regulation that could have also been functional the moment FRP first appeared in cyanobacteria.

One question that remains is why was FRP recruited into the cyanobacterial photoprotection system at all? OCPs that existed before FRP was recruited could recover quickly on their own. Why complicate this functional system? We are aware of two postulated adaptive benefits: first, the OCP1–FRP interaction may offer more sophisticated control of energy use in fast-changing light regimes in the cyanobacterial cell^13^. OCP-mediated photoprotection systems without FRP can only be regulated on the level of messenger RNA transcripts, which act only slowly on a return from stressful to normal light conditions, whereas control by FRP allows potentially faster posttranslational regulation^32^. Second, it may afford superior photoprotection in high light conditions: OCP2 and OCPx paralogues recover so fast that they struggle to stably accumulate the red form at room temperature^13^. OCP1’s more stable red state may then be useful when large amounts of active OCP^R^ are needed, but this high stability may come at the expense of being unable to recover alone. In this scenario, the recruitment of FRP would have enabled the evolution of an ultimately more efficient photoprotection mechanism. However, the interaction could also be an example of non-adaptive complexity that simply became difficult to lose^33^: the acquisition of FRP may have enabled OCP1 to ‘forget’ how to recover efficiently on its own. Once it had lost this ability, FRP would have become essential for full OCP1 function.

The specific compatibility of the FRPL from the *Desulfobacteraceae* species with cyanobacterial OCPs is entirely accidental, because this protein evolved in a genome that contains no OCP. This proves that highly complementary protein surfaces can evolve completely by chance, and that such initially accidental interactions can become incorporated into the biology of organisms. Our work thus raises the possibility that some or even many protein–protein interactions are initially created without the action of direct natural selection. Organisms may in fact be bombarded with virtually fully formed interactions that are created when horizontal transfer, changes in cellular localization or spatiotemporal expression patterns bring together proteins with fortuitously compatible surfaces. From this pool, natural selection would then purge those that are harmful, fix those that are useful and ignore those that are harmless.

## Methods

### Molecular phylogenetics and ancestral sequence reconstruction

To infer the phylogenetic tree of cyanobacterial OCP proteins, we used the OCP dataset of Muzzopappa et al.^16^, and profile-aligned the corresponding amino acid sequences of the three described OCP types therein (OCP1, OCP2, OCPx), using MUSCLE (v.3.8.31)^34^. We added sequences of either cyanobacterial CTD-like homologue proteins (CTDHs) or cyanobacterial HCPs as the respective outgroup. Alignments were corrected manually, sites corresponding to linage-specific insertions and duplicated sequences were removed. Full alignments are in Supplementary Data 1. We used RaxmlHPC-AVX (v.8.2.10)^35^ in the PROTGAMMAAUTO mode to identify the best-fit model of amino acid evolution, which was the Revised Jones–Taylor–Thornton substitution matrix (JTTDCMut)^36^ with empirical base frequencies and gamma distribution of among site rate-variation. We used PhyML (v.3.1)^37^ with SPR moves to infer two ML phylogenies with either CTDH or HCP sequences included, and rooted the trees between either of those sequences and all OCP sequences on our trees. The two phylogenies show basically the same topology, but unassigned grade *A* is first branching on the HCP outgroup tree (Extended Data Fig. 2). As Gloeobacteria, which are known to be early branching cyanobacteria^18–21^, only feature OCPx, but no OCP homologues of the unassigned grades, we used the CTDH outgroup tree for further analyses (Extended Data Fig. 1). The robustness of each topology was tested by running 100 non-parametric bootstraps, and additionally calculating aLRT statistics with PhyML. The ancestral OCP sequences were reconstructed at the internal node on the CTDH outgroup tree, as indicated in Fig. 1b and Extended Data Fig. 1, using marginal reconstruction in the CodeML module of PAML (v.4.9)^38^ with the JTTDCMut substitution model and 16 gamma categories. Ancestral sequences were cropped following parsimony rules and contain the states with the highest posterior probabilities (PP) at all sites selected. The average PP values for all reconstructed proteins are in Extended Data Fig. 3b–e. The ‘altAll’ alternative sequences for every reconstructed ancestor comprises the state with the second highest PP if that state has PP > 0.20, and the ML state otherwise.

For the FRP(L) phylogenetic tree (Fig. 2a), we gathered amino acid sequences using online BLASTP^39^ on 23 February 2022, and the FRP amino acid sequence of *Synechocystis* sp. PCC 6803 (SYNY3) as a query. To specifically find FRPL sequences, we excluded cyanobacteria (taxid:1117) and repeated the search against SYNY3 FRP and subsequently against *P. borbori* FRPL or explicitly searched in taxonomic groups other than cyanobacteria. Additionally, we added metagenomic sequences from the Global Microbial Gene Catalog (GMGC, v.1.0)^40^. Sequences were aligned with MUSCLE (v.3.8.31). The alignment was corrected manually, sites corresponding to linage-specific insertions and duplicated sequences were removed. The full alignment is in Supplementary Data 1. We used RaxmlHPC-AVX (v.8.2.10) in the PROTGAMMAAUTO mode using the Akaike information criterion to identify the best-fit model of amino acid evolution, which was the Le-Gascuel substitution matrix^41^ with fixed base frequencies and gamma distribution of among site rate-variation. We inferred the ML phylogeny, and tested the robustness of the topology by running 100 non-parametric bootstraps. TBEs were calculated with the BOOSTER web tool^42^. Furthermore, aLRT statistics were calculated with PhyML (v.3.1). The tree was rooted between acidobacteria and proteobacteria in the FRPL group and suggests a HGT from an ancestral delta-proteobacterium into an ancestral cyanobacterium. The full tree is in Supplementary Fig. 1. Ancestral FRPL and ancestral FRP sequences (FRPLpreHGT and FRPpostHGT, respectively) were reconstructed at the internal nodes of the tree using marginal reconstruction in the CodeML module of PAML (v.4.9) with the Le-Gascuel substitution matrix (LG) model and 16 gamma categories. Gaps were assigned using parsimony. For the ancestors we resurrected, we chose the amino acid state with the highest PP at each site. The average PP for the reconstructed proteins are in Extended Data Fig. 8b,c.

For the gene tree–species tree reconciliation, we identified all sequences on our FRP(L) tree that could certainly be assigned to a distinct bacterial strain that is also deposited at the Genome Taxonomy Database (GTDB)^43^ with its set of 120 single copy marker protein sequences, using BLASTP^39^. With these aligned, concatenated amino acid sequences, we inferred a ML phylogenetic tree using IQ-Tree 2 (v.2.2)^44^ (-m LG, -b 100, -alrt 1,000), and rooted with acidobacteria as described above. We accordingly inferred a gene tree with FRP and FRPL sequences of the corresponding species, and ran 100 non-parametric bootstraps for this subset. Reconciliation was performed using ML estimation with ALEml_undated in ALE^45^ and the rooted species phylogeny as well as the FRP(L) bootstrap trees as the input. Reconciled trees and ALE output are deposited in the source data.

To reconstruct the alternative ancestral FRPL and alternative ancestral FRP sequences (altFRPLpreHGT and altFRPpostHGT, respectively), we used an initial alignment with fewer sequences in total. The full alignment is in Supplementary Data 1. An ML phylogenetic tree with 100 non-parametric bootstraps was inferred, and the alternative ancestral FRPL and alternative ancestral FRP sequences were reconstructed accordingly at the internal node of that tree, shown in Extended Data Fig. 8e and Supplementary Fig. 2, using marginal reconstruction in the CodeML module of PAML (v.4.9) with the Le-Gascuel substitution matrix substitution model and 16 gamma categories. TBE were calculated with the BOOSTER web tool. Alternative ancestral sequences were cropped following parsimony rules and contain the states with the highest PP at all sides selected. The average PP for the reconstructed proteins are in Extended Data Fig. 8f,g.

For the phylogenetic species tree of OCP-containing cyanobacteria, we identified all sequences on our OCP tree that could certainly be assigned to a distinct cyanobacterial strain that is also deposited at the GTDB with its set of 120 single copy marker protein sequences. As an outgroup, we added sequence sets of closely related malainabacteria as well as sets of more distantly related *Chloroflexota* species. We used these concatenated amino acid sequences, aligned them, and inferred a phylogenetic tree using RaxmlHPC-AVX (v.8.2.10) in the PROTGAMMAAUTO mode, using the Akaike information criterion to identify the best-fit model of amino acid evolution, which was the Le-Gascuel substitution matrix^41^ with empirical base frequencies and gamma distribution of among site rate-variation. We inferred the ML phylogeny, and tested the robustness of the topology by running 100 non-parametric bootstraps. We rooted the tree between cyanobacteria and the outgroup, and mapped the appearance of *frp* and *ocp* genes in corresponding genomes, on the basis of BLASTP and tBLASTn^39^ hits, next to the tree (Extended Data Fig. 6). Assignment of particular OCP sequences to an OCP paralogue group is based on the position of their translated amino acid sequences on our OCP tree (Extended Data Fig. 1).

### Cloning and protein purification

DNA sequences of ancestral OCPs, extant OCP1 from *Synechocystis* sp. PCC 6803 (SYNY3) and FRP (SYNY3) were codon optimized for expression in *E. coli*, and synthesized by either Genscript Biotech or Life Technologies (GeneArt). Synthesized constructs were flanked by *BamHI* and *NotI* cleaving sites for cloning into a modified pRSFDuet-1 vector (Merck Millipore), which encodes a specific human rhinovirus (HRV) 3 C protease cleavage site (LEVLFQ/GP) and a 6xHis tag at the N terminus (resulting plasmid termed pRSFDuetM). After cleavage, all constructs started with GPDPATM. For expression of extant FRP (SYNY3 gene *slr1964*), the pRSFDuetM-FRP vector was transformed into *E. coli* BL21 (DE3) (New England Biolabs), which were grown overnight at 37 °C in Luria–Bertani (LB) medium (1% tryptone, 1% NaCl, 0.5% yeast extract, pH 7.0), supplemented with kanamycin (Kan, 50 µg ml^−1^). The following day, 1 l of LB + Kan was inoculated with 10 ml of overnight culture, and incubated at 37 °C until an optical density (OD_600nm_) of 0.6–0.8, then induced by 0.5 mM isopropyl-β-d-thiogalactopyranoside (IPTG) and grown in a shaking incubator for 24 h at 30 °C. Cells were gathered at 10,000*g* for 10 min, and stored at −20 °C until use. For expression of OCPs (extant OCP1, SYNY3 gene *slr1963* and ancestral OCPs), the corresponding pRSFDuetM-OCPxx constructs were transformed into echinenone-producing *E. coli* BL21 (DE3), harbouring a p25crtO plasmid. The expressions were carried out in 1 l of LB, supplemented with chloramphenicol (34 µg ml^−1^) and Kan (50 µg ml^−1^), which was inoculated by 10 ml of overnight culture, and grown in a shaking incubator at 37 °C until OD_600nm_ = 0.6–0.8. After induction with 0.5 mM IPTG, cells were incubated at 25 °C for 72 h, and finally collected at 10,000*g* for 10 min and stored at −20 °C until use. For purification, frozen cell pellets were resuspended in phosphate-buffered saline (PBS) (137 mM NaCl, 2.7 mM KCl, 12 mM phosphate, pH 7.4), supplemented with 100 mg of lysozyme (Ovobest) and protease inhibitor (1 mM benzamidine, 1 mM ε-amino-caproic acid). Cell lysis was performed by using a FrenchPress (G. Heinemann) in three cycles at 18,000 psi. Afterwards, cell debris was pelleted at 18,000*g* for 15 min at 4 °C. Supernatant was loaded on a 5 ml Co^2+^-HiTrap Talon crude column (Cytiva) using a peristaltic pump. Elution was carried out with imidazole-containing buffer (1× PBS + 350 mM imidazole, pH 7.4), supplemented with HRV 3C protease in a total mass ratio of 500:1 (protein to protease) and dialysed at 4 °C in 3C protease buffer (20 mM Tris, 100 mM NaCl, 2 mM dithiothreitol, pH 8.5) for 18 h. Protein solution was reloaded on a Co^2+^-HiTrap Talon crude column while this time, flow through was collected. In case of FRP, purification was performed by SEC for polishing, while OCP purification was continued with hydrophobic interaction chromatography (HIC) to remove apo-protein. Collected OCP flow-throughs were dialysed overnight in HIC buffer (500 mM (NH_4_)_2_SO_4_, 100 mM urea, 5 mM phosphate, pH 7.5) at 4 °C. HIC was performed on a HiPrepTM 16/10 Phenyl HP column (Cytiva) in an automated Azura FPLC system (Knauer). Proteins were eluted with a hydrophilic buffer (100 mM urea, 5 mM phosphate, pH 7.5). Carotenoid-rich protein fractions were concentrated using 10 kDa molecular weight cut-off (MWCO) centrifugal filter units (Pall Corporation) for SEC. FRP was concentrated with 3 kDa MWCO centrifugal filter units. Then, 500 µl of each concentrated protein solutions were loaded on a SuperdexTM 200 Increase 10/300 column (Cytiva) and eluted with 1× PBS. Proteins were stored at −80 °C until use.

Codon-optimized sequences coding for extant FRPL, ancestral FRP, and ancestral FRPL proteins were obtained from Integrated DNA Technologies (IDT) or Twist Biosciences. They were cloned into pET-LIC vectors containing an N- or C-terminal 6xHis tag using Gibson Assembly Master Mix (New England Biolabs). The oligonucleotides used are shown in Supplementary Table 1. Correct assembly was verified by Sanger Sequencing (Microsynth). Plasmids were transformed into *E. coli* BL21 (DE3) (Invitrogen). For protein overproduction, 50 ml of LB, supplemented with carbenicillin (Carb) (100 μg ml^−1^), were inoculated with a single colony from a fresh LB + Carb plate, and grown overnight at 37 °C in a shaking incubator. Six lots of 500 ml of LB + Carb were inoculated with overnight cultures at OD_600nm_ = 0.01, and grown to OD_600nm_ = 0.6–0.8 for roughly 2.5 h. Protein overproduction was induced with 1 mM IPTG. After 4 h, cells were gathered at 4,392*g* for 20 min at 4 °C and cell pellets were stored at −20 °C until usage. For purification, cells were resuspended in 35 ml of buffer A (300 mM NaCl, 20 mM Tris, 20 mM imidazole, 5 mM β-mercaptoethanol, pH 8.0), and one tablet of cOmplete Protease Inhibitor Cocktail (Roche) was added. Cells were disrupted twice in an LM10 microfluidizer (Microfluidics) at 13,000 psi. Lysate was cleared by centrifugation at 29,930*g* for 30 min, and being passed through a 0.45 µm syringe filter, then loaded on a 5 ml Bio-Scale Mini Nuvia Ni-charged IMAC Cartridge (BioRad). After washing with 25 ml of buffer A, protein was eluted with a linear gradient over 20 ml from 0 to 100% of buffer B (300 mM NaCl, 20 mM Tris, 500 mM imidazole, 5 mM β-mercaptoethanol, pH 8.0) in an NGC system (BioRad). Fractions containing the protein were verified on in-house casted 15% SDS gels, and were pooled for SEC with a HiLoad 26/600 Superdex column (Cytiva) in SEC buffer (200 mM NaCl, 20 mM KCl, 20 mM HEPES, pH 7.5) in an NGC system. Purity of the fractions containing the protein were verified on in-house casted 15% SDS gels, and were pooled for concentration at 2,000*g* with Amicon Ultra centrifugal filter units (Millipore) with a MWCO of 3 kDa. Proteins were stored at −20 °C until usage.

### Carotenoid extraction and ultra-fast liquid chromatography analysis

To analyse the carotenoid content of OCP holo-proteins, 50 µl of concentrated protein solution was mixed with 1 ml of acetone and centrifuged at maximum speed at 4 °C to spin down precipitated protein. Yellowish supernatant was evaporated in a centrifugal vacuum concentrator (Eppendorf) at 30 °C until the acetone evaporated completely and carotenoids had precipitated as red crystals. Remaining water solution was removed, and red carotenoid crystals were redissolved in 50 µl of acetone. The carotenoid-rich solution was transferred into a sample vial that was placed in an UFLC NexeraX2 system (Shimadzu), equipped with an Accucore C30 column (Thermo Fisher Scientific, 250 × 2.1 mm, 2.6 µm particle size, 150 Å pore size). As mobile phase eluents, buffer A (methanol to water, 95:5) and buffer B (methanol to THF, 7:3) were used with the following protocol: 0–4.3 min 0% of buffer B, 4.3–8.6 min linear gradient from 0 to 100% of buffer B, 8.6–15.6 min 100% of buffer B, 15.6–20.1 min 0% of buffer B with a constant flow rate of 0.4 ml min^−1^. Eluted carotenoids were verified by mass spectrometry to correlate elution times with specific carotenoid species as well as by thin-layer chromatography and comparison with reference samples.

### Ultraviolet–visible spectroscopy and kinetic analysis

Absorption spectra were recorded with a Maya2000Pro spectrometer (Ocean Optics), coupled via a fibre to a deuterium tungsten light source (Sarspec) and a cuvette holder (CVH100, Thorlabs). For OCP/FRP kinetic analyses, a temperature-controlled cuvette holder with a constant stirring device qpod2e (Quantum Northwest) was fibre-coupled to a CCS100/M spectrometer (Thorlabs) and a SLS201L/M tungsten light source (Thorlabs). For illumination with actinic light, a 3 W light-emitting diode (Avonec) with a maximum emission at 455 nm was used. Different OCP^O^ (mixed with different extant or ancestral FRP or extant or ancestral FRPL in various molar ratios, or alone) were photo-switched into the red state (OCP^R^) by applying blue light for at least 3 min and 30 s or until a plateau was reached, and photo-recovery was constantly followed at 550 nm after turning off the blue light source. Recovery time constants (*τ*) were determined by fitting relaxation curves of the OCP^R^ to OCP^O^ back-conversions with a mono-exponential decay function and standard deviations (s.d.) of three independent replicates were calculated.

### Circular dichroism spectroscopy

Far-ultraviolet circular dichroism spectroscopy was used to assess the secondary structure of heterologously produced *P. borbori* FRPL (PbFRPL) in solution. The protein was diluted to a concentration of roughly 50 µg ml^−1^ in circular dichroism Buffer (100 mM NaF, 10 mM Na_2_HPO_4_/NaH_2_PO_4_, pH 7.5), and was measured in a 0.1 cm cuvette at room temperature using a JASCO J-810 spectropolarimeter (Jacso) in the range of 190–240 nm in 0.2 nm scanning steps. Three successive spectra were recorded, baseline corrected and averaged.

### Native mass spectrometry

FRPL protein sample from *P. borbori* (PbFRPL) was stored at −20 °C before being buffer exchanged into 200 mM ammonium acetate (pH 6.8) by multiple rounds of concentration and dilution using Pierce protein concentrators (Thermo Fisher). The sample was then diluted to 4 µM  (monomer) immediately before the measurements. Data were collected using in-house gold-plated capillaries on a Q Exactive mass spectrometer (ThermoFisher Scientific), operated in positive ion mode with a source temperature of 100 °C and a capillary voltage of 1.2 kV. In-source trapping was set to −100 V to help with the dissociation of small ion adducts. Ion transfer optics and voltage gradients throughout the instruments were optimized for ideal transmission. Spectra were acquired with ten micro-scans to increase the signal-to-noise ratio with transient times of 64 ms, corresponding to the resolution of 17,500 at *m*/*z* = 200, and AGC target of 1.0 × 10^6^. The noise threshold parameter was set to three and the scan range used was 350 to 8,000 *m*/*z*.

### X-ray crystallography

Crystallization of *P. borbori* FRPL (PbFRPL) was performed by the hanging-drop method at 20 °C in 2 µl drops, consisting of equal amounts of protein and precipitation solutions. PbFRPL crystallized at 119 µM within 20 days in 0.2 M Li_2_SO_4_, 0.1 M CHES, pH 9.5 and 1.4 M sodium:potassium tartrate. Before data collection, crystals were flash-frozen in liquid nitrogen without the use of cryo-protectants. Synchrotron data were collected under cryogenic conditions at the P13 beamline, operated by the European Molecular Biology Laboratory (EMBL) Hamburg at the PETRA III storage ring (Deutsches Elektronen Synchrotron)^46^. Data were integrated and scaled with XDS, and merged with XSCALE^47^. Structures were determined by molecular replacement with PHASER^48^, manually built in COOT^49^ and refined with PHENIX^50^. For structure determination by molecular replacement, the crystal structure of FRP from *Synechocystis* sp. PCC 6803 (PDB ID 4JDX, ref. ^25^) was used as a search model. The final structure of PbFRPL was uploaded to the RCSB PDB under accession number 8AG8. Data were rendered and visualized with PyMol (v.2.4.0)^51^.

### Whole-genome nanopore sequencing

After several rounds of cultivation, we re-sequenced the whole genome of *P. borbori* to rule out *frpl* gene loss on cultivation (a possible explanation for absence of FRPL in all model organisms), plasmid localization (that could facilitate HGT) or sample contamination, but found the genome to be a single, circular chromosome of 5.34 MB in size, entailing one copy of the *frpl* gene, but no OCP, HCP or CTDH homologues (Extended Data Fig. 7d). Genomic DNA of stationary phase *P. borbori* was obtained using the NucleoBond HMW DNA kit (Macherey-Nagel) according to the manufacturer’s guidelines, and using lysozyme for cell lysis (final concentration 1 mg ml^−1^) for 1 h at 37 °C in 2 ml of 10 mM Tris-HCl, pH 8.0. DNA quality and concentration were assessed via NanoDrop 8000 spectrophotometer and Qubit 3 fluorometer using double-stranded DNA BR reagents. Library preparation was performed using the Ligation Sequencing Kit SQK-LSK109 (Oxford Nanopore Technologies), according to the manufacturer’s guidelines, except the input DNA was increased fivefold to match the molarity expected in the protocol as no DNA shearing was applied. Sequencing was performed on a MinION Mk1B device for 24 h using a ‘Flongle Flow Cell’ (FLO-FLG001, cell chemistry R9.4.1). Nanopore data were base-called with ONT Guppy base-calling software. Long reads were assembled using canu^52^, resulting in a single circular chromosome. Raw reads are deposited at the National Center for Biotechnology Information (NCBI) Sequence Read Archive and can be accessed under BioProject no. PRJNA865569 and BioSample accession no. SAMN30120905.

### Cultivation and genetic manipulation of *P. borbori*

The type stain DSM17834 of the delta-proteobacterium *P. borbori* was purchased from the German Collection of Microorganisms and Cell Cultures (Braunschweig, Germany). It was cultivated aerobically in PME medium (0.5% peptone, 0.3% meat extract, pH 7.0) at 28 °C, and a growth curve of biological triplicates was recorded. The generation time (*G*) during exponential growth was estimated using the formula $G = \frac{{{{\Delta }}t}}{{3.3\log \left( {\frac{{{\mathrm{OD}}_2}}{{{\mathrm{OD}}_1}}} \right)}}$.

Protein fusions for in vivo localization with epi-fluorescence microscopy were generated by PCR amplification of the *frpl* gene of *P. borbori* including 200 bp of the 5′ untranslated region and insertion into pSG1164 vectors with an N- or C-terminal mVenus coding sequence and a ‘GGGGGSL’ linker sequence in frame using Gibson Assembly Master Mix (NEB). Correct assembly was verified by Sanger Sequencing (Microsynth). Chemically competent *P. borbori* were prepared by modification of a protocol by Irani and John^53^, initially developed for *P. aeruginosa*, as follows: the medium was changed to PME, and temperatures were lowered to 28 °C. Plasmids were transformed into *P. borbori* following the transformation protocol of Irani and John^53^, but changing the heat shock temperature to 30 °C, the medium to PME, the growth temperature to 28 °C and the carbenicillin concentration to 100 µg ml^−1^. Plates were incubated at 28 °C for 48 h until colonies were visible.

### Epi-fluorescence microscopy

For epi-fluorescence microscopy, *P. borbori* cells were grown at 28 °C and 200 r.p.m. to OD_600_ = 0.6 for ‘exponential growth’ and for 2 days to OD_600_ of around 1.0 for ‘starvation’ conditions in PME media. Cells were fixed on 1% agarose pads by sandwiching 100 µl of melted agarose between two coverslips (12 mm, Menzel). Then 3 µl of the culture was added onto a round coverslip (25 mm; Marienfeld) and fixed with an agarose pad. For widefield image acquisition, a Zeiss Observer A1 microscope (Carl Zeiss) with an oil immersion objective (×100 magnification, 1.45 numerical aperture, alpha Plan-FLUAR; Carl Zeiss) was used with a charge-coupled-device camera (CoolSNAP EZ; Photometrics) and an HXP 120 metal halide fluorescence illumination with intensity control. For epi-fluorescence microscopy, a green fluorescent protein filter set was used (BrightLine 470/40, Beamsplitter 495 and Brightline 525/50). Samples were illuminated for 0.5 to 2 s at mid-cell plane. Whole-cell integrated fluorescence was determined per cell and corrected for background fluorescence. Final editing of images was done in ImageJ2/ FIJI (v.1.52)^54,55^.

### Analytical SEC

Analytical SEC was performed with a Superdex 75 Increase 3.2/300 column (Cytiva), equilibrated with 1× PBS at a flow rate of 0.1 ml min^−1^ and a total sample injection volume of 20 µl. For measuring at blue light illumination, four 3 W LEDs (Avonec) with an emission maximum at 455 nm were mounted on a 20 cm heat sink at constant distances in front of the SEC column to continuously illuminate the sample on the column. Absorption was recorded at 280, 496 and 550 nm to follow elution profiles.

### AlphaFold2 protein complex prediction

AlphaFold2 protein complex models were generated using the ColabFold server^56^ on 20 May 2022, using as input sequences the CTD of either OCP1 from *Synechocystis* sp. PCC 6803 (SYNY3) or AncOCPall and FRP (SYNY3) with default settings. Further, the structure of full-length AncOCPall was predicted separately. On 3 November 2022, we repeated the analysis with the CTD of OCPx from *G. kilaueensis* JS1 or an S264Y mutant (serine at position 264 (SYNY3 numeration) was changed to tyrosine) of that OCPx with FRP (SYNY3). Modelled structures are deposited in the source data. Data were rendered and visualized with PyMol (v.2.4.0)^51^.

### Native PAGE

Native PAGE was performed in a Mini-Protean Tetra Cell (Biorad) by using in-house casted gradient gels with 3–14% acrylamide concentration in a Tris-glycine buffer system without SDS to obtain native protein conditions. No stacking gel was used. The electrophoresis chamber was constantly cooled in a fridge and illuminated by four 3 W LEDs (Avonec) with an emission maximum at 455 nm to photo-switch the OCP proteins in-gel. The voltage was set to 80 V constantly for 240 min, and subsequently to 120 V for another 100 min.

### Reporting summary

Further information on research design is available in the Nature Portfolio Reporting Summary linked to this article.

## Supplementary information

Supplementary Information Supplementary Figs. 1–3, Table 1 and Discussion.

Reporting Summary

Supplementary Data 1 Underlying multiple sequence alignments of Extended Data Figs. 1, 2 and 6 and Supplementary Figs. 1 and 2.

## Data Availability

Source data are available at the Open Research Data Repository of the Max Planck Society (Edmond) under the 10.17617/3.44RHFZ. Crystallography data are available at RCSB PDB under accession number 8AG8. Sequencing data are available on NCBI Sequence Read Archive under BioProject PRJNA865569.
