# Supplementary material for: Fortuitously compatible protein surfaces primed allosteric control in cyanobacterial photoprotection
Source: Nat Ecol Evol. 2023 Apr 3;7(5):756–67. doi: 10.1038/s41559-023-02018-8 (PMC10172135; doi:10.1038/s41559-023-02018-8)
Supplement: Supplementary file 1 — Supplementary Figs. 1–3, Table 1 and Discussion. [file 41559_2023_2018_MOESM1_ESM.pdf]

nature ecology & evolution

Article

https://doi.org/10.1038/s41559-023-02018-8

Fortuitously compatible protein surfaces primed allosteric control in cyanobacterial photoprotection

In the format provided by the authors and unedited

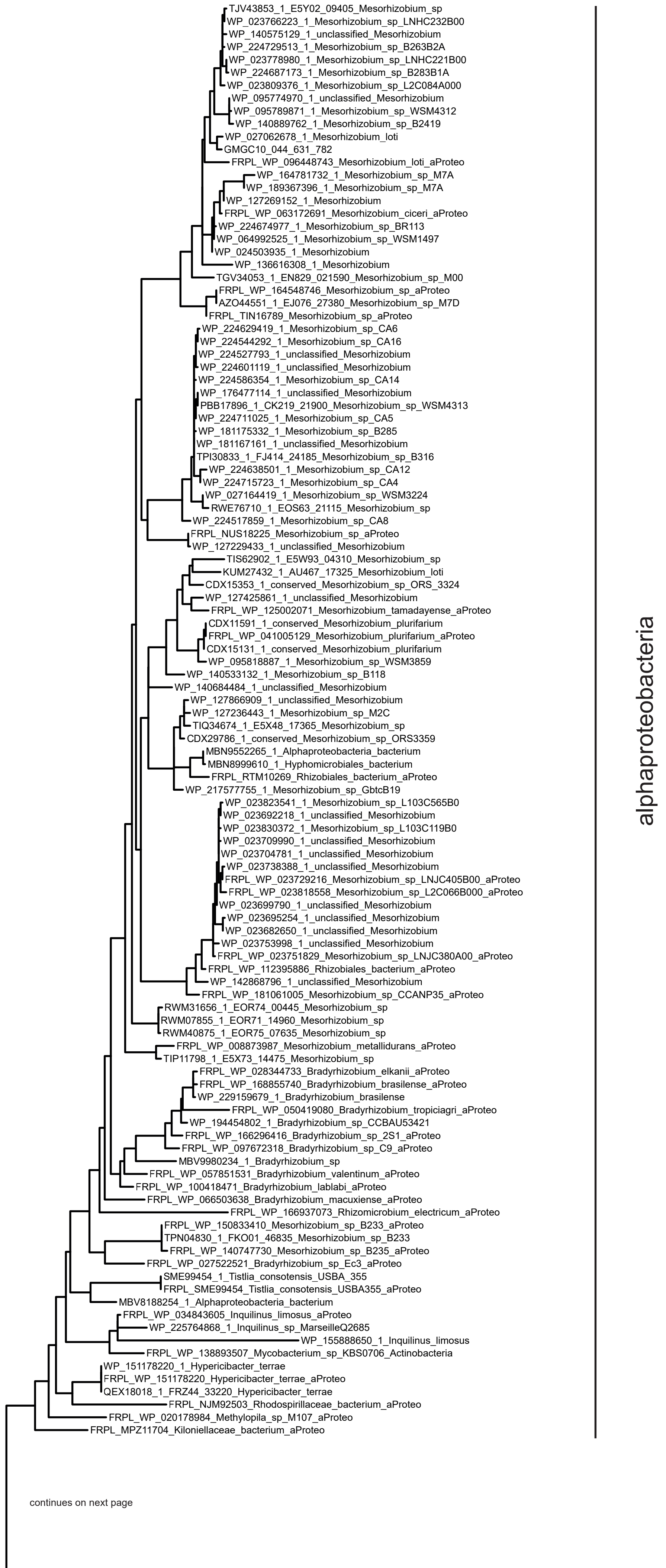

continues on next page

continued from previous page

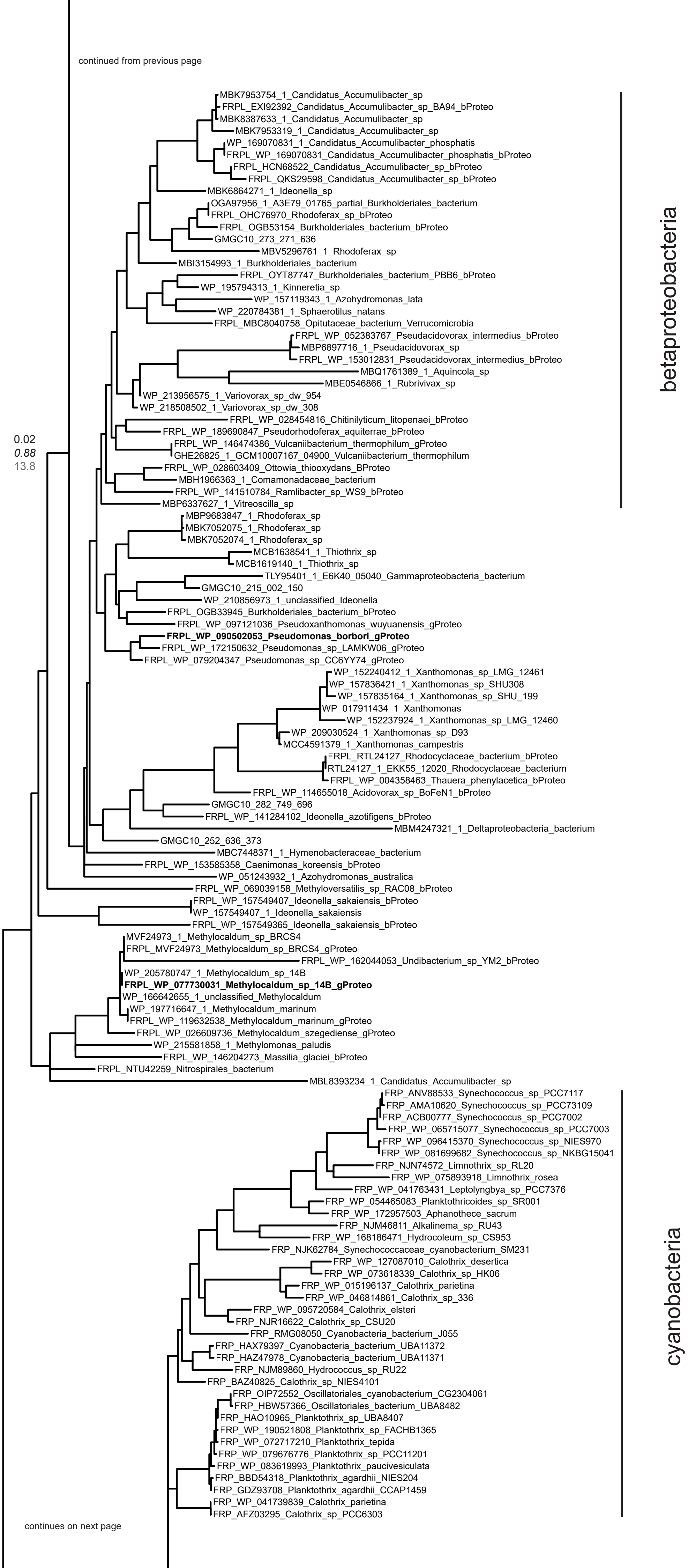

continues on next page

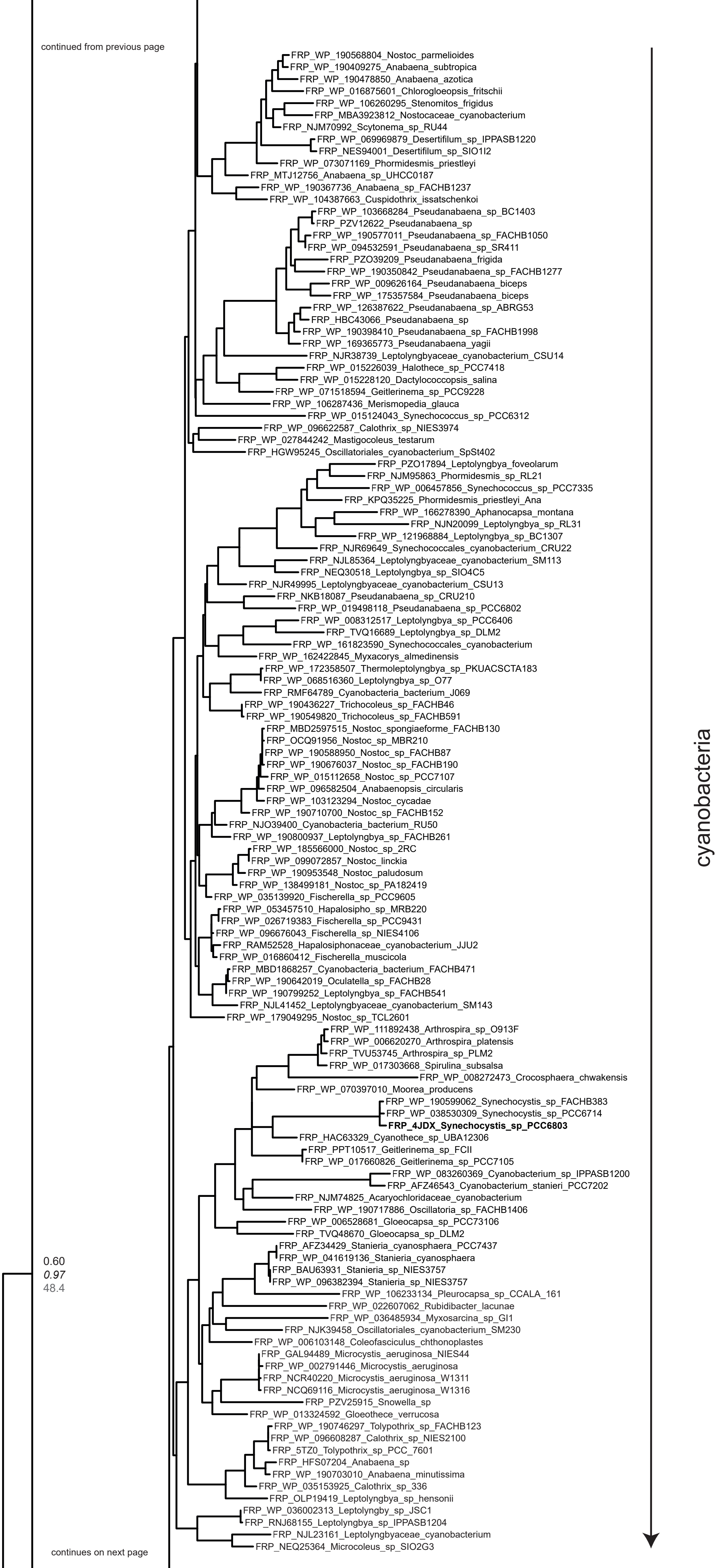
continued from previous page
FRP_WP_190568804_Nostoc_parmelioides
FRP_WP_190409275_Anabaena_subtropica
FRP_WP_190478850_Anabaena_azotica
FRP_WP_016875601_Chlorogloeopsis_fritschii
FRP_WP_106260295_Stenomitos_frigidus
FRP_MBA3923812_Nostocaceae_cyanobacterium
FRP_NJM70992_Scytonema_sp_RU44
FRP_WP_069969879_Desertifilum_sp_IPPASB1220
FRP_NES94001_Desertifilum_sp_SIO1I2
FRP_WP_073071169_Phormidesmis_priestleyi
FRP_MTJ12756_Anabaena_sp_UHCC0187
FRP_WP_190367736_Anabaena_sp_FACHB1237
FRP_WP_104387663_Cuspidothrix_issatschenkoi
FRP_WP_103668284_Pseudanabaena_sp_BC1403
FRP_PZV12622_Pseudanabaena_sp
FRP_WP_190577011_Pseudanabaena_sp_FACHB1050
FRP_WP_094532591_Pseudanabaena_sp_SR411
FRP_PZO39209_Pseudanabaena_frigida
FRP_WP_190350842_Pseudanabaena_sp_FACHB1277
FRP_WP_009626164_Pseudanabaena_biceps
FRP_WP_175357584_Pseudanabaena_biceps
FRP_WP_126387622_Pseudanabaena_sp_ABRG53
FRP_HBC43066_Pseudanabaena_sp
FRP_WP_190398410_Pseudanabaena_sp_FACHB1998
FRP_WP_169365773_Pseudanabaena_yagii
FRP_NJR38739_Leptolyngbyaceae_cyanobacterium_CSU14
FRP_WP_015226039_Halothece_sp_PCC7418
FRP_WP_015228120_Dactylococcopsis_salina
FRP_WP_071518594_Geitlerinema_sp_PCC9228
FRP_WP_106287436_Merismopedia_glauca
FRP_WP_015124043_Synechococcus_sp_PCC6312
FRP_WP_096622587_Calothrix_sp_NIES3974
FRP_WP_027844242_Mastigocoleus_testarum
FRP_HGW95245_Oscillatoriales_cyanobacterium_SpSt402
FRP_PZO17894_Leptolyngbya_foveolarum
FRP_NJM95863_Phormidesmis_sp_RL21
FRP_WP_006457856_Synechococcus_sp_PCC7335
FRP_KPQ35225_Phormidesmis_priestleyi_Ana
FRP_WP_166278390_Aphanocapsa_montana
FRP_NJN20099_Leptolyngbya_sp_RL31
FRP_WP_121968884_Leptolyngbya_sp_BC1307
FRP_NJR69649_Synechococcales_cyanobacterium_CRU22
FRP_NJL85364_Leptolyngbyaceae_cyanobacterium_SM113
FRP_NEQ30518_Leptolyngbya_sp_SIO4C5
FRP_NJR49995_Leptolyngbyaceae_cyanobacterium_CSU13
FRP_NKB18087_Pseudanabaena_sp_CRU210
FRP_WP_019498118_Pseudanabaena_sp_PCC6802
FRP_WP_008312517_Leptolyngbya_sp_PCC6406
FRP_TVQ16689_Leptolyngbya_sp_DLM2
FRP_WP_161823590_Synechococcales_cyanobacterium
FRP_WP_162422845_Myxacorys_almedinensis
FRP_WP_172358507_Thermoleptolyngbya_sp_PKUACSCTA183
FRP_WP_068516360_Leptolyngbya_sp_O77
FRP_RMF64789_Cyanobacteria_bacterium_J069
FRP_WP_190436227_Trichocoleus_sp_FACHB46
FRP_WP_190549820_Trichocoleus_sp_FACHB591
FRP_MBD2597515_Nostoc_spongiaeforme_FACHB130
FRP_OCQ91956_Nostoc_sp_MBR210
FRP_WP_190588950_Nostoc_sp_FACHB87
FRP_WP_190676037_Nostoc_sp_FACHB190
FRP_WP_015112658_Nostoc_sp_PCC7107
FRP_WP_096582504_Anabaenopsis_circularis
FRP_WP_103123294_Nostoc_cycadae
FRP_WP_190710700_Nostoc_sp_FACHB152
FRP_NJO39400_Cyanobacteria_bacterium_RU50
FRP_WP_190800937_Leptolyngbya_sp_FACHB261
FRP_WP_185566000_Nostoc_sp_2RC
FRP_WP_099072857_Nostoc_linckia
FRP_WP_190953548_Nostoc_paludosum
FRP_WP_138499181_Nostoc_sp_PA182419
FRP_WP_035139920_Fischerella_sp_PCC9605
FRP_WP_053457510_Hapalosipho_sp_MRB220
FRP_WP_026719383_Fischerella_sp_PCC9431
FRP_WP_096676043_Fischerella_sp_NIES4106
FRP_RAM52528_Hapalosiphonaceae_cyanobacterium_JJU2
FRP_WP_016860412_Fischerella_muscicola
FRP_MBD1868257_Cyanobacteria_bacterium_FACHB471
FRP_WP_190642019_Oculatella_sp_FACHB28
FRP_WP_190799252_Leptolyngbya_sp_FACHB541
FRP_NJL41452_Leptolyngbyaceae_cyanobacterium_SM143
FRP_WP_179049295_Nostoc_sp_TCL2601
FRP_WP_111892438_Arthrospira_sp_O913F
FRP_WP_006620270_Arthrospira_platensis
FRP_TVU53745_Arthrospira_sp_PLM2
FRP_WP_017303668_Spirulina_subsalsa
FRP_WP_008272473_Crocosphaera_chwakensis
FRP_WP_070397010_Moorea_producens
FRP_WP_190599062_Synechocystis_sp_FACHB383
FRP_WP_038530309_Synechocystis_sp_PCC6714
FRP_4JDX_Synechocystis_sp_PCC6803
FRP_HAC63329_Cyanothece_sp_UBA12306
FRP_PPT10517_Geitlerinema_sp_FCII
FRP_WP_017660826_Geitlerinema_sp_PCC7105
FRP_WP_083260369_Cyanobacterium_sp_IPPASB1200
FRP_AFZ46543_Cyanobacterium_stanieri_PCC7202
FRP_NJM74825_Acaryochloridaceae_cyanobacterium
FRP_WP_190717886_Oscillatoria_sp_FACHB1406
FRP_WP_006528681_Gloeocapsa_sp_PCC73106
FRP_TVQ48670_Gloeocapsa_sp_DLM2
FRP_AFZ34429_Stanieria_cyanosphaera_PCC7437
FRP_WP_041619136_Stanieria_cyanosphaera
FRP_BAU63931_Stanieria_sp_NIES3757
FRP_WP_096382394_Stanieria_sp_NIES3757
FRP_WP_106233134_Pleurocapsa_sp_CCALA_161
FRP_WP_022607062_Rubidibacter_lacunae
FRP_WP_036485934_Myxosarcina_sp_GI1
FRP_NJK39458_Oscillatoriales_cyanobacterium_SM230
FRP_WP_006103148_Coleofasciculus_chthonoplastes
FRP_GAL94489_Microcystis_aeruginosa_NIES44
FRP_WP_002791446_Microcystis_aeruginosa
FRP_NCR40220_Microcystis_aeruginosa_W1311
FRP_NCQ69116_Microcystis_aeruginosa_W1316
FRP_PZV25915_Snowella_sp
FRP_WP_013324592_Gloeothece_verrucosa
FRP_WP_190746297_Tolypothrix_sp_FACHB123
FRP_WP_096608287_Calothrix_sp_NIES2100
FRP_5TZ0_Tolypothrix_sp_PCC_7601
FRP_HFS07204_Anabaena_sp
FRP_WP_190703010_Anabaena_minutissima
FRP_WP_035153925_Calothrix_sp_336
FRP_OLP19419_Leptolyngbya_sp_hensonii
FRP_WP_036002313_Leptolyngby_sp_JSC1
FRP_RNJ68155_Leptolyngbya_sp_IPPASB1204
FRP_NJL23161_Leptolyngbyaceae_cyanobacterium
FRP_NEQ25364_Microcoleus_sp_SIO2G3
cyanobacteria
0.60
0.97
48.4
continues on next page

continued from previous page

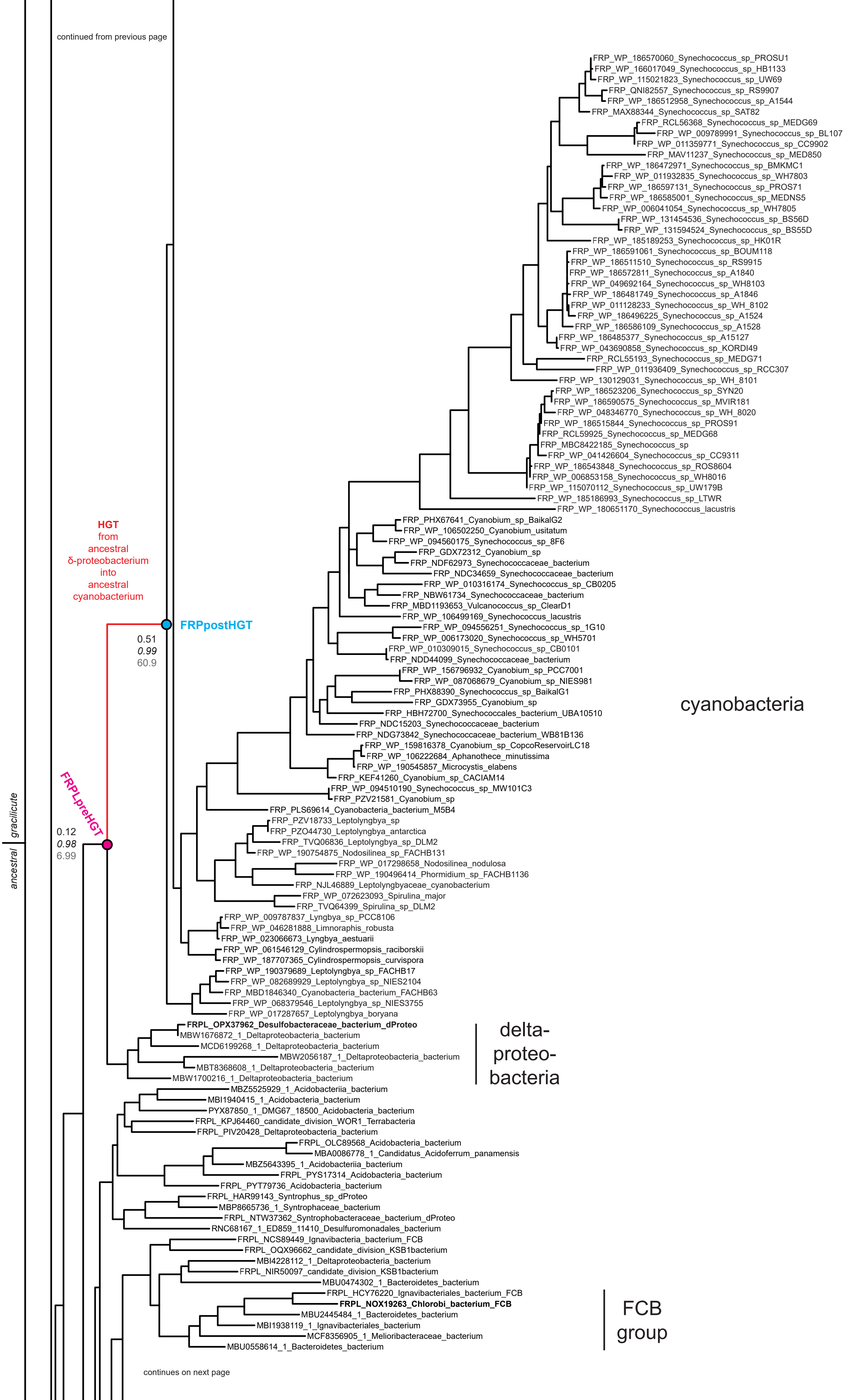

continues on next page

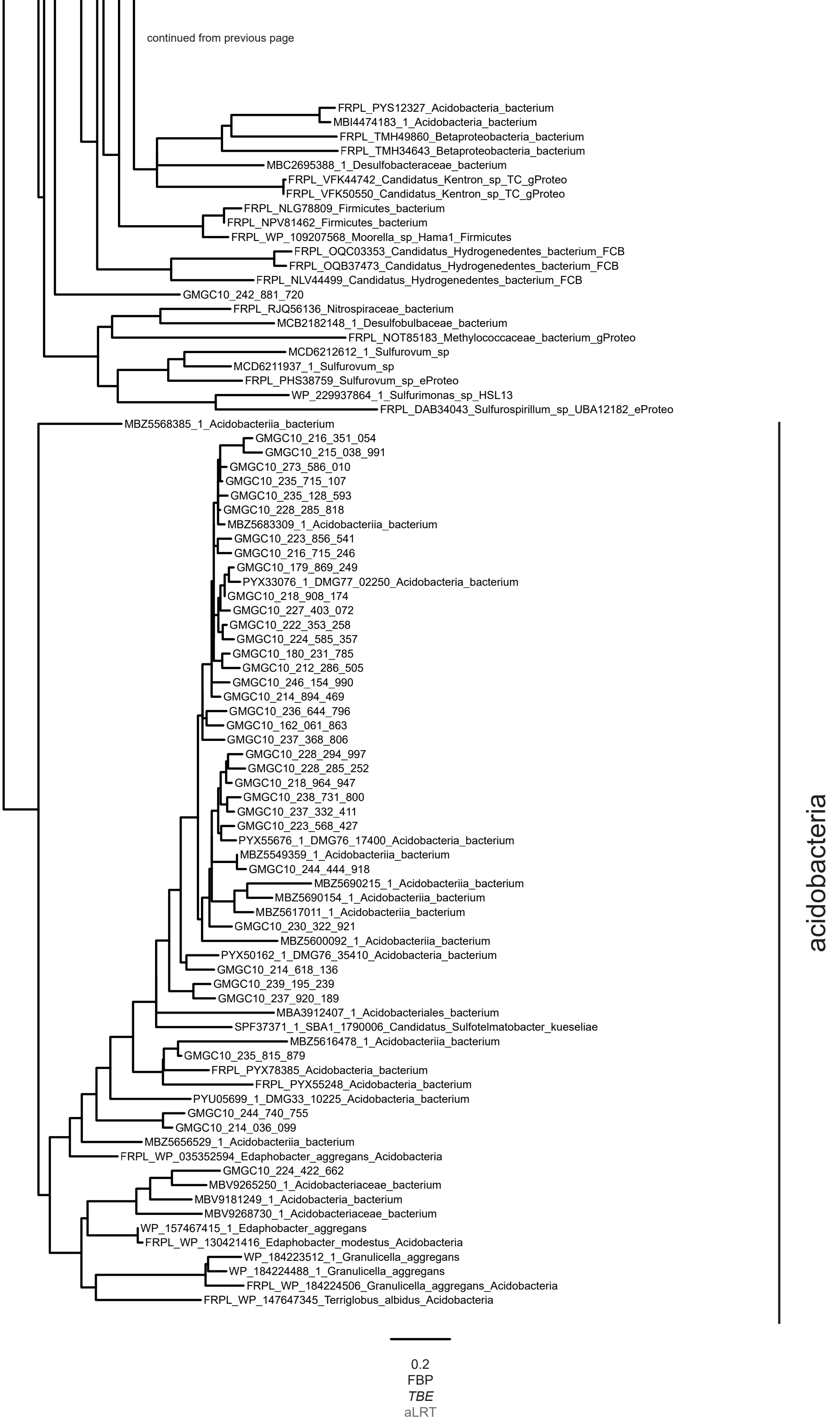

Supplementary Fig. 1| Complete phylogeny of FRP and FRPL proteins. Uncollapsed ML phylogeny of data shown in Fig. 2a with reconstructed ancestral proteins that existed before (FRPLpreHGT) and after the horizontal gene transfer event (FRPpostHGT) at labeled nodes. Felsenstein Bootstrap Probabilities (FBP) and Transfer Bootstrap Expectations (*TBE*) of 100 replicates are given at important nodes. Grey numbers are approximated likelihood ratio test values (aLRT). Branch-lengths represent average substitutions per site. Examined proteins in this study are highlighted in bold. Underlying multiple sequence alignment in Supplementary Data 1.

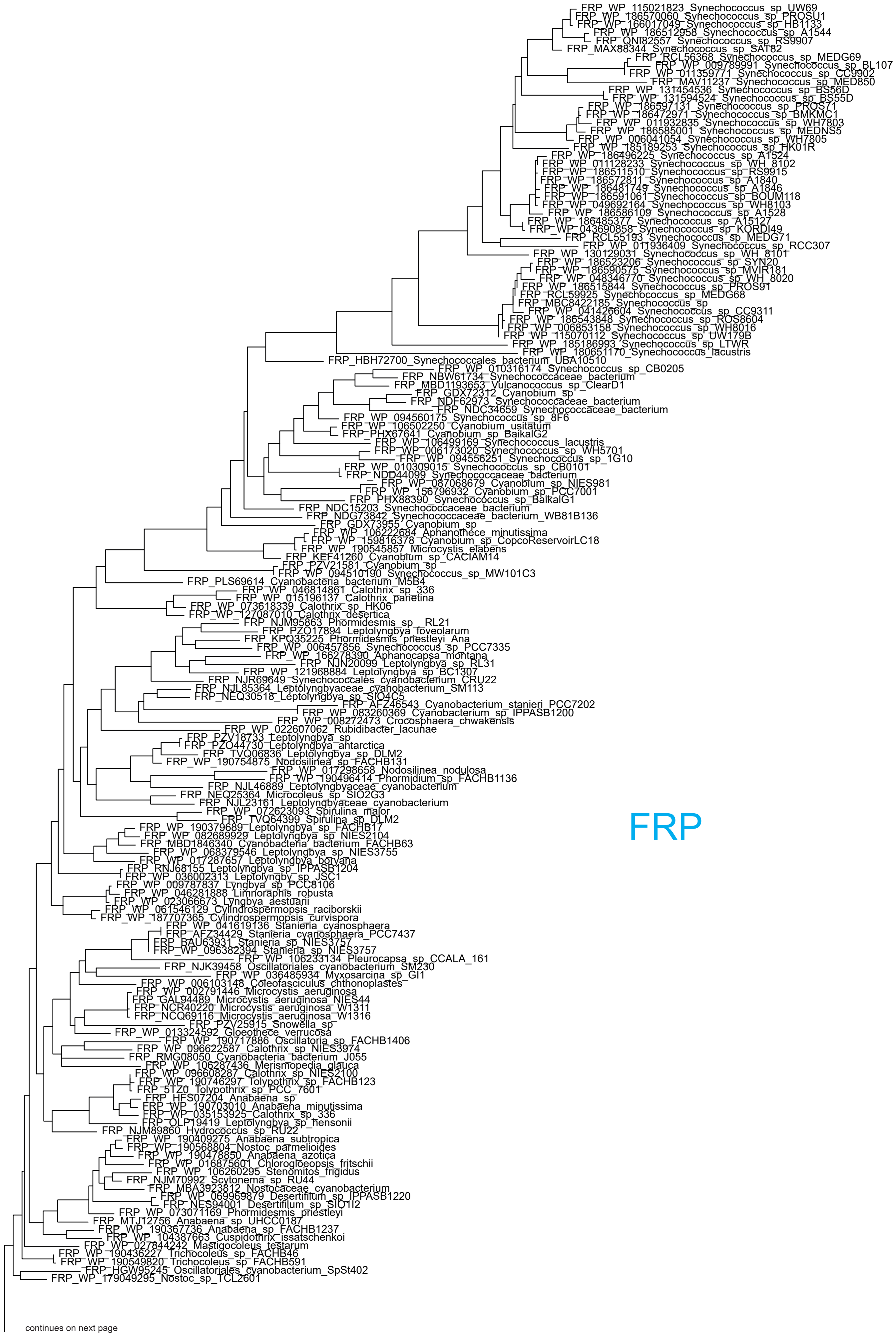

continues on next page

continued from previous page

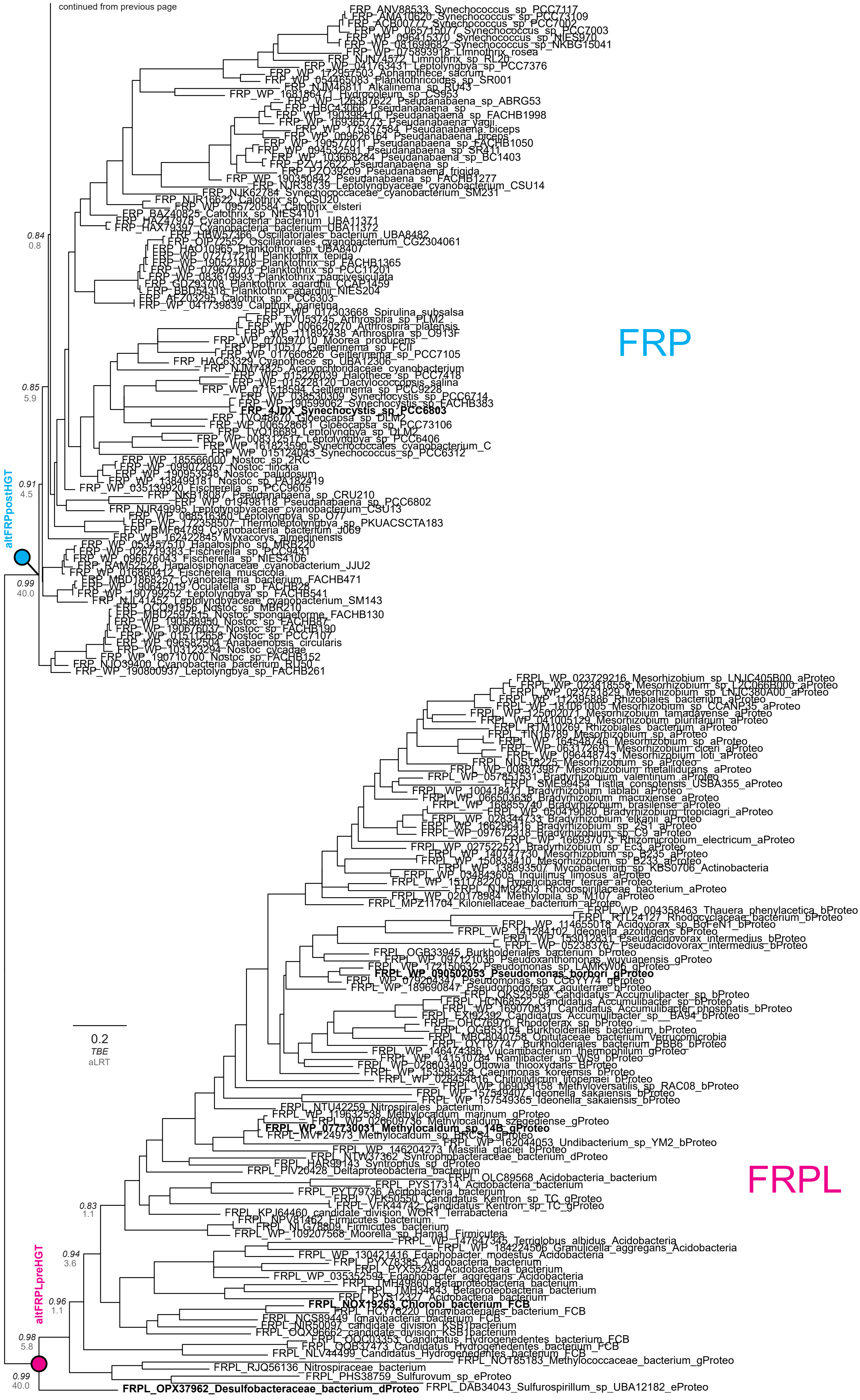

Supplementary Fig. 2| Complete unrooted initial alternative phylogeny of FRP and FRPL proteins. Uncollapsed ML phylogeny of data shown in Extended Data Fig. 8e with reconstructed alternative ancestral proteins that existed before (altFRPLpreHGT) and after the horizontal gene transfer event (altFRPpostHGT) at labeled nodes. Transfer Bootstrap Expectations (*TBE*) of 100 replicates are given in italics at important nodes. Grey numbers are approximated likelihood ratio test values (aLRT). Branch-lengths represent average substitutions per site. Examined proteins in this study are highlighted in bold. Underlying multiple sequence alignment in Supplementary Data 1.

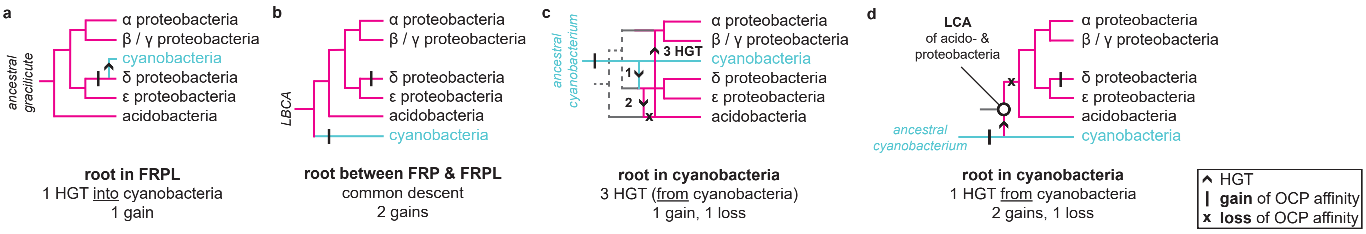

Supplementary Fig. 3| Scenarios of FRP and FRPL evolution. a, Simplified scheme of the evolution of FRP and FRPL proteins, according to the most parsimonious scenario shown in Fig. 2a. **b-d**, Simplified schemes of the alternative, but less parsimonious scenarios. *LBCA*, last bacterial common ancestor. HGT, horizontal gene transfer.

Supplementary Tab. 1| Utilized oligonucleotides in this study

oligonucleotide	sequence (5' to 3')
primer for linearization of pLIC vectors, forward	CTCGAGCACCACCACCAC
primer for linearization of pLIC vectors, reverse	ATGTATATCTCCTTCTTAAAGTTAAACAAAATTATTTCTAGAGG
primer for amplification of genes to clone into pLIC vectors, forward	ATAATTTTGTTTAACTTTAAGAAGGAGATATACAT
primer for amplification of genes to clone into pLIC vectors, reverse	GCCGGATCTCAGTGGTGG

Supplementary Discussion

The fortuitous ability of FRPL to act on OCP is agnostic about an HGT event

We postulate that cyanobacteria acquired FRP via a horizontal gene transfer (HGT) from delta-proteobacteria early in their history after OCP first formed in an ancestral cyanobacterium (Fig. 2a). This is the most parsimonious explanation given the available sequence data our phylogenetic tree is based on, but also implies a large number of gene losses in different kinds of non-cyanobacterial groups (Supplementary Fig. 3a).

A root between FRPs and FRPLs would place the evolution of FRPLs near the last common ancestor (LCA) of all bacteria[23], which would imply even more gene losses in virtually all major bacterial groups (Supplementary Fig. 3b). However, even in this scenario, the intrinsic ability of FRPLs to act on OCP would have evolved in a non-cyanobacterium by chance without direct selective pressure. In fact, FRPLs would have randomly drifted in and out of the sequence space that enables the interaction with OCP during its evolution, and happened to be capable of the interaction when OCP first evolved in cyanobacteria.

We have also considered the possibility that the root may lie within the FRP clade, implying HGT to proteobacteria and acidobacteria. However, we consider this scenario very implausible: it would require a transfer from a relatively modern cyanobacterium with FRP into at least the LCA of all proteobacteria and acidobacteria. This is not only temporally implausible, but further incongruent with the topology of our gene tree: when rooted inside FRPs, our phylogeny does not place proteobacteria and acidobacteria sister to all FRPs. This means our gene tree would require additional HGT between different kinds of proteobacteria to explain the distribution of FRPL (Supplementary Fig. 3c). In addition, this scenario implies that FRPLs in delta-proteobacteria fortuitously retained their ability to interact with OCP since around the time of the LCA of all living cyanobacteria.

Another possibility is that our gene tree is simply incorrect, perhaps owing to the short length of the FRP and FRPL proteins. For example, if the true tree in the FRPL clade actually follows the species phylogeny of acidobacteria and proteobacteria, we could root the tree inside the FRP clade and explain the tree with a single horizontal transfer (Supplementary Fig. 3d). FRP's function would then be ancestral. But this would also imply that the ability to bind OCP was lost many times independently in FRPLs, and was regained in only a small set of delta-proteobacteria. We also consider this very implausible.

Further, a reconciliation of 100 bootstrap trees with a species phylogeny using amalgamated likelihood estimation (ALE)[45] found no root between FRPs and FRPLs, but 72 tree topologies featuring a root within the FRPLs.

Taken together, the OCP-matching interface in FRPLs evolved without direct selective pressure mainly by chance even if we remain agnostic about the horizontal transfer event, that we think is still the most likely scenario here.
