# Supplementary material for: Fortuitously compatible protein surfaces primed allosteric control in cyanobacterial photoprotection
Source: Nat Ecol Evol. 2023 Apr 3;7(5):756–67. doi: 10.1038/s41559-023-02018-8 (PMC10172135; doi:10.1038/s41559-023-02018-8)
Supplement: Supplementary file 2 — Reporting Summary [file 41559_2023_2018_MOESM2_ESM.pdf]

nature portfolio

Corresponding author(s): Georg Hochberg
Thomas Friedrich

Last updated by author(s): Feb 8, 2023

Reporting Summary

Nature Portfolio wishes to improve the reproducibility of the work that we publish. This form provides structure for consistency and transparency in reporting. For further information on Nature Portfolio policies, see our Editorial Policies and the Editorial Policy Checklist.

Statistics

For all statistical analyses, confirm that the following items are present in the figure legend, table legend, main text, or Methods section.

n/a	Confirmed	
☐	☒	The exact sample size (n) for each experimental group/condition, given as a discrete number and unit of measurement
☐	☒	A statement on whether measurements were taken from distinct samples or whether the same sample was measured repeatedly
☐	☒	The statistical test(s) used AND whether they are one- or two-sided<br>*Only common tests should be described solely by name; describe more complex techniques in the Methods section.*
☒	☐	A description of all covariates tested
☒	☐	A description of any assumptions or corrections, such as tests of normality and adjustment for multiple comparisons
☐	☒	A full description of the statistical parameters including central tendency (e.g. means) or other basic estimates (e.g. regression coefficient) AND variation (e.g. standard deviation) or associated estimates of uncertainty (e.g. confidence intervals)
☐	☒	For null hypothesis testing, the test statistic (e.g. F, t, r) with confidence intervals, effect sizes, degrees of freedom and P value noted<br>*Give P values as exact values whenever suitable.*
☒	☐	For Bayesian analysis, information on the choice of priors and Markov chain Monte Carlo settings
☒	☐	For hierarchical and complex designs, identification of the appropriate level for tests and full reporting of outcomes
☒	☐	Estimates of effect sizes (e.g. Cohen's d, Pearson's r), indicating how they were calculated

Our web collection on statistics for biologists contains articles on many of the points above.

Software and code

Policy information about availability of computer code

Data collection	NCBI online BLASTP and tBLASTn (https://blast.ncbi.nlm.nih.gov) were used to collect amino acid sequences from NCBI or GTDB databases.
Data analysis	We used MUSCLE (v3.8.31) for alignments, RaxmlHPC-AVX (v8.2.10), PhyML (v3.1), and IQ-Tree2 (v2.2) to infer phylogenetic trees, and PAML (v4.9) for ancestral sequence reconstruction. TBE were calculated with the BOOSTER (v0.1.2) web tool (https://booster.pasteur.fr). ALE (v1.0) was used for gene tree - species tree reconcilliation. To process crystallophy data, XDS (v01.22), XSCALE (v1.0), PHASER (v1.0), COOT (v0.8), and PHENIX (v1.2) were used. Data was visualized with PyMol (v2.4.0). Sequencing data was processed using ONT Guppy (v5.0.16) and canu (v2.1.1). Editing of microscopic images was done in ImageJ2/ FIJI (v1.52). AlphaFold2 (v.1.3) protein complex models were generated utilizing the ColabFold server (https://colab.research.google.com/github/sokrypton/ColabFold) on May 20, 2022 or October, 3, 2022. Phython (v3.7) was used to visualize data.

For manuscripts utilizing custom algorithms or software that are central to the research but not yet described in published literature, software must be made available to editors and reviewers. We strongly encourage code deposition in a community repository (e.g. GitHub). See the Nature Portfolio guidelines for submitting code & software for further information.

Data

Policy information about availability of data

All manuscripts must include a data availability statement. This statement should provide the following information, where applicable:

- Accession codes, unique identifiers, or web links for publicly available datasets
- A description of any restrictions on data availability
- For clinical datasets or third party data, please ensure that the statement adheres to our policy

Source data is available at the Open Research Data Repository of the Max Planck Society (Edmond) under doi: 10.17617/3.44RHFZ. Crystallography data is available at RCSB Protein Data Base (PDB) under accession number 8AG8. Sequencing data is available on NCBI Sequence Read Archive (SRA) under BioProject PRJNA865569. NCBI, JGI, or GMGC identifiers for amino acid sequences used in this study are provided in Supplementary Data 1.

Human research participants

Policy information about studies involving human research participants and Sex and Gender in Research.

Reporting on sex and gender	This study did not involve human reasearch participants.
Population characteristics	This study did not involve human reasearch participants.
Recruitment	This study did not involve human reasearch participants.
Ethics oversight	This study did not involve human reasearch participants.

Note that full information on the approval of the study protocol must also be provided in the manuscript.

Field-specific reporting

Please select the one below that is the best fit for your research. If you are not sure, read the appropriate sections before making your selection.

☒ Life sciences ☐ Behavioural & social sciences ☐ Ecological, evolutionary & environmental sciences

For a reference copy of the document with all sections, see nature.com/documents/nr-reporting-summary-flat.pdf

Life sciences study design

All studies must disclose on these points even when the disclosure is negative.

Sample size	Sampling of homologs for phylogeny and ancestral sequence reconstruction was performed including all genomes available from NCBI at the time of collection, and processed to minimize redundancy. Biochemical and biophysical experiments were performed on purified stocks of recombinantly expressed and purified proteins. For microscopy analyses, fluorescence data of 28 individual cells per condition were sampled.
Data exclusions	Sequence data was only excluded to minimize redundancy as descibed above. No other data was excluded from the analyses.
Replication	Biochemical and biophysical experiments were replicated at least three times with similar results.
Randomization	Experiments were performed on recombinantly expressed and purified proteins. No group allocations were done in our experiments.
Blinding	Consistent with accepted practice in biochemistry and molecular biology, protein purifications and biophysical measurements were not blinded.

Reporting for specific materials, systems and methods

We require information from authors about some types of materials, experimental systems and methods used in many studies. Here, indicate whether each material, system or method listed is relevant to your study. If you are not sure if a list item applies to your research, read the appropriate section before selecting a response.

Materials & experimental systems

n/a	Involved in the study	
☒	☐	Antibodies
☒	☐	Eukaryotic cell lines
☒	☐	Palaeontology and archaeology
☐	☒	Animals and other organisms
☒	☐	Clinical data
☒	☐	Dual use research of concern

Methods

n/a	Involved in the study	
☒	☐	ChIP-seq
☒	☐	Flow cytometry
☒	☐	MRI-based neuroimaging

Animals and other research organisms

Policy information about studies involving animals; ARRIVE guidelines recommended for reporting animal research, and Sex and Gender in Research

Laboratory animals	The study did not involve laboratory animals.
Wild animals	The study did not involve wild animals.
Reporting on sex	The study did not involve any animals or plants.
Field-collected samples	The study did not involve samples collected from the field.
Ethics oversight	This study did not involve animals. No ethics approvance or guidance were required.

Note that full information on the approval of the study protocol must also be provided in the manuscript.
